# The Legacy of the TTASAAN Report – Premature Conclusions and Forgotten Promises About SPECT Neuroimaging: A Review of Policy and Practice Part II

**DOI:** 10.3389/fneur.2022.851609

**Published:** 2022-05-17

**Authors:** Dan G. Pavel, Theodore A. Henderson, Simon DeBruin, Philip F. Cohen

**Affiliations:** ^1^PathFinder Brain SPECT, Deerfield, IL, United States; ^2^The International Society of Applied Neuroimaging (ISAN), Denver, CO, United States; ^3^The Synaptic Space, Inc., Denver, CO, United States; ^4^Neuro-Luminance, Inc., Denver, CO, United States; ^5^Dr. Theodore Henderson, Inc., Denver, CO, United States; ^6^Neuro-Laser Foundation, Denver, CO, United States; ^7^Good Lion Imaging, Baltimore, MD, United States; ^8^Nuclear Medicine, Lions Gate Hospital, Vancouver, BC, Canada; ^9^Department of Radiology, University of British Columbia, Vancouver, BC, Canada

**Keywords:** SPECT, procedure, Parkinsonian, traumatic brain injury, differential diagnosis, comorbidity

## Abstract

Brain perfusion single photon emission computed tomography (SPECT) scans were initially developed in 1970s. A key radiopharmaceutical, hexamethylpropyleneamine oxime (HMPAO), was not stabilized until 1993 and most early SPECT scans were performed on single-head gamma cameras. These early scans were of inferior quality. In 1996, the Therapeutics and Technology Assessment Subcommittee of the American Academy of Neurology (TTASAAN) issued a report regarding the use of SPECT in the evaluation of neurological disorders. This two-part series explores the policies and procedures related to perfusion SPECT functional neuroimaging. In Part I, the comparison between the quality of the SPECT scans and the depth of the data for key neurological and psychiatric indications at the time of the TTASAAN report vs. the intervening 25 years were presented. In Part II, the technical aspects of perfusion SPECT neuroimaging and image processing will be explored. The role of color scales will be reviewed and the process of interpreting a SPECT scan will be presented. Interpretation of a functional brain scans requires not only anatomical knowledge, but also technical understanding on correctly performing a scan, regardless of the scanning modality. Awareness of technical limitations allows the clinician to properly interpret a functional brain scan. With this foundation, four scenarios in which perfusion SPECT neuroimaging, together with other imaging modalities and testing, lead to a narrowing of the differential diagnoses and better treatment. Lastly, recommendations for the revision of current policies and practices are made.

## Introduction

In 1996, the Therapeutics and Technology Assessment Subcommittee of the American Academy of Neurology (TTASAAN) issued a report regarding the use of perfusion single photon emission computed tomography (SPECT) in functional brain imaging (1). While the authors of the TTASAAN report did not intend this to be the definitive position of perfusion SPECT neuroimaging (in the text, they mandated periodic revision as the field advanced), this 1996 report, nonetheless, has stood and remains to stand as the final word on perfusion SPECT neuroimaging in Neurology and Psychiatry since. As a result, neurologists and psychiatrists have distanced themselves or outright disparaged SPECT neuroimaging and its role in the evaluation of a patient. Allegorically, there were no smartphones in 1996. Rather there were brick-like mobile phones with external antenna. In 2000, the first touchscreen became available and integrated cameras became available in 2002. Finally, in 2007, the I-phone was unveiled ushering in the era of the smart phone. Relying on the TTASAAN report today is equivalent to relying on an assessment of modern cell phone technology and applications based on 1996 technology. Just as the smartphone computer in your hand bears little resemblance to the bulky, heavy, dialing devices of old, modern SPECT neuroimaging bears little resemblance to its 1996 predecessor. In 1996, there were only single-headed gamma cameras, unstable radiotracers, no quantitative software, no normative databases, no advanced image reconstruction software, no iterative reconstruction, no CT hybrid imagers, no solid-state detectors, and no artificial intelligence algorithms. All of these advances have radically changed and improved perfusion SPECT neuroimaging. Furthermore, the research literature encompassing over 60,000 patients across multiple neurological and neuropsychiatric disorders did not exist (as reviewed extensively in Part I of this two-part series).

This two-part series explores the policies and procedures related to perfusion SPECT functional neuroimaging and, ultimately, makes recommendations for revisions to the current policies and practices. In Part I, the comparison between the quality of the SPECT scans and the depth of the data for key neurological and psychiatric indications at the time of the TTASAAN report vs. the intervening 25 years were presented (2). We reviewed the research literature on traumatic brain injury (TBI) (encompassing over 24,000 subjects) and showed SPECT perfusion imaging is more sensitive that CT or MRI for detecting TBI. We rebutted many criticisms of detecting TBI with perfusion SPECT, and demonstrated that perfusion SPECT meets the criteria for a Type A recommendation based on the criteria set forth in the TTASAAN report. We reviewed the use of perfusion SPECT in epilepsy, including the findings that ictal-interictal SPECT has an accuracy of 85.9% in localizing seizure foci. The vast field of neuroimaging in dementia was reviewed and perfusion SPECT was shown to have 96% sensitivity and 80% specificity in differentiating Alzheimer's disease (AD) from fronto-temporal dementia (FTD), comparable to fluorodeoxyglucose-positron emission tomography (FDG-PET) (2, 3). Similarly, the conversion from mild cognitive impairment (MCI) to AD or FTD can be predicted with both perfusion SPECT and FDG-PET. FDG-PET was found to predict the conversion from MCI to AD with a sensitivity of 70–90% and a specificity of 82.4–90%, while perfusion SPECT scans predict the conversion from MCI to AD with a sensitivity ranging from 89 to 97% and a specificity of 89–100% (2). In addition, we reviewed the SPECT neuroimaging research findings in neurotoxicity, attention-deficit hyperactivity disorder, depression, bipolar disorder, obsessive compulsive disorder, and stroke.

In Part II, the technical aspects of perfusion SPECT neuroimaging and the process of interpreting a SPECT scan will be presented.

### Limitations in Functional Neuroimaging

Interpretation of a functional brain scan requires not only anatomical knowledge, but also technical understanding on how to correctly perform a scan. This is no less true for positron emission tomography (PET) or functional magnetic resonance imaging (fMRI) than it is for SPECT scanning. Our intent here is not to paint a rosy picture of perfusion SPECT imaging at the expense of other modalities. Rather, we merely wish to remind the field that PET, diffusion tensor imaging (DTI), and fMRI have limitations, as well.

The usefulness of any imaging technique, as well as its widespread adoption into clinical practice and clinical research is ultimately dependent upon the consistency, rigor, and quality of the methodology used to create the images. An MRI from a 0.5 Tesla magnet is quite different from that of a 3 Tesla magnet. Moreover, the technique of obtaining the MRI image has significant effects upon the resulting image. If the sequences for T2 weighted imaging, proton-density weighted, or DTI are not programmed correctly and motion is not controlled, then the resulting images can be uninterpretable.

While technically not functional neuroimaging, DTI is being studied as a method of detecting TBI. DTI is a highly sophisticated sequence of MRI imaging. Yet, there is not an agreed upon protocol for obtaining DTI images. As a result, the quality, accuracy, and clinical significance of DTI imaging varies greatly across facilities. This has led to conflicting results for certain conditions and a lack of uniformity in the field (4, 5). According to some experts, while DTI may be a sensitive measure, currently it lacks the level of specificity necessary for application in clinical practice (5). Similarly, fMRI has suffered from a lack of unified protocols.

Functional neuroimaging is particularly vulnerable to technical errors or flaws. As discussed in Part I (2), an analysis of the validity of fMRI post-processing methods has revealed significant flaws which potentially invalidate many fMRI studies and the resulting conclusions about fMRI findings in certain conditions (6). The American Psychiatric Association has questioned the value of fMRI research into psychiatric disorders (7).

An important distinction often lost in the textbook or the lecture hall concerning neuroimaging modalities is the distinction between ***resolution*** and ***contrast***. While anatomical MRI and of CT (to a lesser extent) have superior resolution (1 mm), FDG-PET scans have a resolution of 5 mm and SPECT using standard sodium iodide crystals have a resolution close to 10 mm. On the other hand, contrast is the ability to discern an abnormal signal from background. The sensitivity of CT for detecting contrast agents is in the millimolar range, while that of MRI is in the micromolar range. The sensitivity of SPECT neuroimaging for detecting a radiopharmaceutical is in the nanomolar range, exceeding MRI by a thousand-fold and exceeding CT by a million-fold (8–10). This is perhaps best illustrated with seizure imaging, wherein CT and MRI show no abnormality, while perfusion SPECT can reliably localize the seizure focus or foci. (11–19). Similarly, as reviewed extensively in Pavel and colleagues (2), CT and MRI often miss areas of cortical dysfunction following concussion or TBI [e.g., Figure 4 in (2)], while perfusion SPECT can readily detect TBI and differentiate TBI from control with a >95% accuracy (20, 21).

Modern fMRI has limited resolution and contrast (6) with pixel size generally 2–3 mm (22), as discussed in Part I. In PET imaging, it is important to recall that PET scanners visualize the annihilation of a positron, not the release of a positron from a tracer. In contrast to gamma radiation emitters (as used in SPECT) wherein the point of gamma photon release corresponds to the exact site at which the radiopharmaceutical is bound or retained; positron emitters have a degree of inaccuracy related to the physics of positrons and the range that they travel before annihilation (23–25). For example, the range of ^18^F is up to 6 mm (26, 27) and the range of ^18^O is up to 10 mm (27). In the case of ^18^F, this can lead to a 3.5% degradation of resolution for soft tissue, such as brain (27).

On the other hand, resolution in many cases is a red herring that matters very little in the pragmatic clinical practice. For example, the resolution of amyloid PET imaging is immaterial. If amyloid is present in the grey matter, then the scan is considered positive for Alzheimer's disease. If it is negative, then Alzheimer's disease is unlikely (caveats discussed below). The scan can be read from across the room. Similarly, a ^123^I-ioflupane scan (DaTscan) has relatively low resolution, but the pattern is distinctive. If the tracer is absent from the striatal “tail,” then the scan reveals degeneration of the dopamine system in the striatum.

In the interpretation of perfusion SPECT scans compared to FDG PET scans, the resolution differences of modern versions of these modalities matters very little. As described in depth in Part I (2), both modalities can visualize the posterior cingulate gyrus, the parietal cortices, and the temporal cortices and render, for example, a diagnosis of Alzheimer's disease with similar accuracy (89 vs. 89%) (2, 3, 28, 29).

In part II of this Policy and Practice Review, we will address the technical aspects of perfusion SPECT functional neuroimaging which can gravely affect the quality of the scan. We will describe future changes which are now months to years away. We will also articulate best practices for obtaining, processing, and interpreting a perfusion SPECT scan. Lastly, we will discuss the integration of perfusion SPECT scans into coordinated and insightful utilization of multiple neuroimaging modalities. This integrative philosophy will be illustrated with four clinical examples. These technical aspects, together with the aforementioned extensive review of perfusion SPECT findings in a number of conditions (2), will guide recommendations for changes in policy and practice.

## Technical Aspects of SPECT Neuroimaging

Perfusion SPECT functional neuroimaging is no less vulnerable to degradation resulting from poor or flawed technique. As has been laid out in detail in Part I (2), the SPECT images of the early 1990s which were the basis of the TTASAAN committee's decisions and report, are technically far removed from the current high-quality perfusion SPECT images we consistently work with today. Nonetheless, technique still varies widely from facility to facility. Hence, the process of interpreting a perfusion SPECT scan necessitates an appreciation of neuroanatomy, the effects of altered anatomy, camera properties, acquisition methods, reconstruction and filtering algorithms, and attenuation correction. It is time to have a closer look at the technical considerations of SPECT instrumentation and processing that contribute to the execution of high-quality brain SPECT scans.

### The Gamma Camera

**Figure 1:**
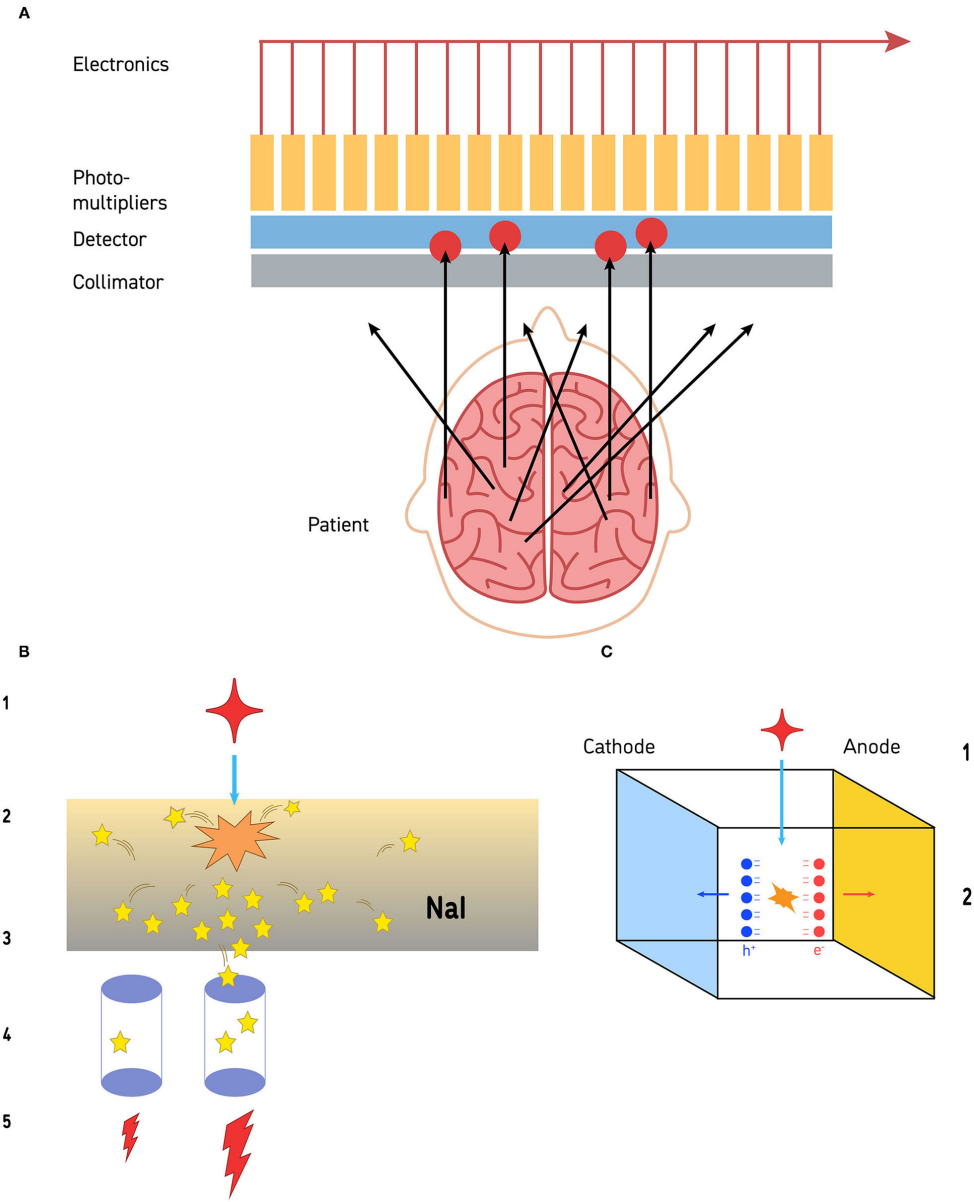
**(A)** The collimation process is illustrated. Gamma photons released by the radiopharmaceutical travel away from the site of emission in all directions. Those that impact the collimator at an angle cannot pass through. Gamma photons that reach the collimator in alignment with the channels of the collimator can pass through and reach the detector (a sodium iodide crystal in this illustration). The limit to resolution is the ***size of the photomultipliers***, distance from the structure of interest, and the resolution of the collimator. **(B)** Detection of gamma photons by sodium iodide crystal detectors is a multi-step process. The gamma photon (red star −1) strikes the sodium iodide crystal (orange starburst) and a burst of visible light photons (yellow stars) is released (2). The light photons radiate in all directions (3). Some of those visible light photons will impact on photodetectors (purple cylinders −4). Photodetectors closer to the site of gamma photon impact will receive more visible light photons, while those further away from the site will receive less (4). Those photodetectors that receive more visible light photons will release a greater electrical current, while the detectors further away will release a small electrical current (5). This is the basis of the Anger circuitry for geometric localization. **(C)** Cadmium-zinc-telluride (CZT) detectors are solid-state and directly convert gamma photons into electrons at room temperature. When a gamma photon (1) strikes a CZT detector (2), pairs of electrons and holes are generated (red and blue balls, respectively). These create an electrical potential across the detector which is proportional to the energy of the impinging radiation. The resolution is limited by the size of the CZT detector—currently about 2.4 mm on a side.

Most commercial gamma cameras today are based on a scintillating sodium-iodide (Na-I) crystal, coupled to an array of photomultiplier tubes (PMTs). The field of view of the camera is given by the size of the crystal. A gamma photon released in the radioactive decay of the radiopharmaceutical that reaches the crystal, causes it to emit a brief flash of light which scatters in all directions, with a strength proportional to the energy of the gamma radiation (Figure 1). The light is detected, amplified, and converted into an electrical pulse by the PMTs. The amount of light measured by each PMT depends on its location in reference to the origin of the light flash. The PMT closest to the event location produces a greater electrical pulse compared to the surrounding PMTs. The exact location is determined by a center of mass calculation according to the methodology developed by Hal Anger. Even though the typical PMT has a diameter of 2 in. or 3 in, the Anger principle gives the gamma camera an intrinsic resolution on the order of 4 mm at 140 keV (the gamma energy of ^99m^-Technetium, the most common radionuclide in SPECT imaging).

To obtain useful images, the incoming angles of incidence of gamma photons must be restricted. For this purpose, a collimator is positioned between the patient (the source of radiation) and the crystal (the detector). A collimator is made of a perforated slab of lead, absorbing most gamma photons except those which arrive parallel to the axis of the collimator holes (Figure 1). The design parameters of the collimator (e.g., hole diameter and length, septa thickness), determine the overall resolution of the imaging system. The commonly used collimator for ^99m^-Technetium studies is the studies is the low energy/high resolution (LEHR) collimator with a resolution of ~6.5 mm at 10 cm distance. Note: the resolution of the collimator is a function of distance; thus, to maximize image quality, the distance between the patient and the surface of the collimator must be minimized.

As the collimator rejects most of the incoming radiation, it shall be clear that it has a huge impact on the overall system sensitivity. Specialized collimator geometries have been developed over the years such as fan beam or cone beam but their prevalence in the field is small.

The size of the detector determines the efficiency with which a particular nuclear medicine procedure can be executed. For a general-purpose gamma camera, the most common detector size is 21 in. × 15 in. allowing a whole-body bone scan (one of the most frequently ordered SPECT scans) to be performed in a single pass. Obviously, this size is much more than what is needed for a brain scan and complicates patient positioning as the large physical size of the detectors make it harder to get close to the patient's head without truncating parts of the brain. These pragmatic limitations result in the brain being a greater distance from the detector and therefore occupying a smaller percentage of the detector's field of view. Hence, fewer counts accumulate in each cell of the matrix (see Acquisition Matrix and Acquisition Zoom below) and many cells have no counts whatsoever.

One way to increase gamma camera sensitivity is by using more detectors. The most common gamma camera today has 2 detectors, cutting imaging time in half for most procedures (or doubling the statistics compared to a single detector camera). In many cameras, these heads can be positioned in opposition to each other or at a right angle to each other. In the past, triple detector cameras were manufactured specifically for brain imaging, but small production volumes meant that such products were not viable, and manufacture of these cameras has ceased. Nonetheless, surviving triple detector cameras are still widely used.

A fundamental advance in gamma camera technology has been underway over the past decade and new cameras with higher resolution and higher efficiency are now commercially available. Essentially, gamma photon detectors have changed little since the early days of research. The workhorse of the gamma camera has been the sodium iodide (NaI) crystal described above (Figure 1B).

In contrast, the recently developed Cadmium Zinc Telluride (CZT) detector is a semiconductor that directly converts x-ray or gamma-ray photons into electrons at room temperature. Typical CZT detectors are fabricated with a thin layer of metal deposited on both of surfaces of the detector to act as electrodes. These electrodes then allow the detectors to be electrically biased and, thus, creating an electrical potential across the detector. One surface acts as a cathode and one acts as an anode. As shown in Figure 1C, when a gamma photon collides with the biased CZT, a pair of electron/hole is generated, which are proportional to the energy of the absorbed radiation. Negatively charged electrons and positively charged holes migrate to their respective electrodes and are collected. This process is referred to as photoelectric absorption.

CZT detectors have led to the development of cameras with much higher resolution. The resolution is no longer limited by the spread of visible light photons in the NaI crystal or by the collimator. CZT detectors can be made very small (e.g., 2.4 mm square). There are 1,000 CZT detectors in each camera head. Each functions essentially as a pin-hole camera. As a result, the intrinsic spatial resolution (ISR) of a modern CZT gamma camera falls to 2.46 mm. This is considerably higher than the 7.0 mm ISR of current dual-head gamma cameras and exceeds the 4.0 mm ISR of currently available PET cameras.

### Patient Preparation and Positioning

Equally critical in obtaining a quality SPECT scan is the proper preparation of the patient. This will be briefly explored here, but a detailed description is provided in the Canadian Association of Nuclear Medicine guidelines (30). Patient preparation begins long before the day of the scan and includes potentially stopping medications. The decision to stop current medications prior to a scan should never be made lightly. Some patients or referring clinicians may prefer to obtain a scan without medications; however, scans are still informative if patients do not stop their current medications—with a few exceptions. The decision should be made in consultation with the referring or treating physician. If the decision is made to stop medications, then a safe and comfortable taper should be planned.

There are a small number of medications which should always be stopped prior to a functional brain scan, because they either artificially increase or decrease brain perfusion. A number of commonly ingested substances increase cerebral blood flow. For example, all stimulant medications should be withheld for 72 h prior to a scan. Patients should avoid caffeine for 48 h prior to a scan. Similarly, over-the-counter medications or supplements containing pseudoephedrine, caffeine, ephedrine, guarana, or taurine, as well as energy drinks, should be avoided. Nicotine should be avoided for 48 h, but this is difficult for most individuals who use nicotine. Therefore, a modified restriction for 12 h prior to the scan is often acceptable. Acetazolamide (Diamox) is used to treat glaucoma and high altitude sickness. This medication robustly increases cerebral blood flow and should be stopped 48 h prior to a scan. Substances that artificially lower cerebral blood flow should be avoided as well. Benzodiazepines should be withheld for 48 h prior to a scan. If a patient is on a stable dose of a benzodiazepine, they may require a careful taper to discontinue the medication or it may be necessary to accept a low dose of benzodiazepine, if the risk of withdrawal or seizures is too great. Alcohol and marijuana, as well as any illicit drugs, should be avoided for 48 h before a functional brain scan. Caution should be exercised with patients who are heavy users of alcohol, caffeine, nicotine or illicit drugs to not precipitate a dangerous withdrawal situation.

Patients should be cautioned to avoid chewing gum or eating in the 2 h prior to injection of the radiopharmaceutical for a perfusion SPECT scan. This reduces extraneous uptake of tracer into salivary glands. Similarly, keeping patients calm and limiting situations that induce weeping will reduce uptake in lacrimal glands. Patients should be well hydrated prior to a SPECT scan.

At the time of radiopharmaceutical injection, the patient should be positioned in a comfortable reclining chair or exam table with raised head and an IV started. Ideally, lights should be dimmed and the room quiet. Sound-dampening headphones can be helpful. For a baseline scan, the patient should be asked to close their eyes at the time of injection of the radiopharmaceutical and keep them closed for 2 mins. Closing the eyes reduces visual cortex activity. Regardless of whether ^99m^Tc- ethyl cysteinate dimer (ECD) or ^99m^Tc- hexamethylpropyleneamine oxime (HMPAO) is utilized, the activity of the brain will be captured, essentially a frozen distribution of radiopharmaceutical, within 2 mins of injection.

Scrupulous technique must be utilized in quality control and preparation of the radiopharmaceutical. The details of this process extend beyond the scope of this article. Suffice to say, it is critical not to spill radiopharmaceutical on the patient or the patient's clothing near the head.

Following radiopharmaceutical injection, the patient should have ~40 mins to allow for washout of non-specific binding. During this time, the patient should be encouraged to drink at least 16–24 ounces (500–750 ml) of water and to void urine at least once. This facilitates comfort during the scanning process, reduces motion, and eliminates excess unbound radiopharmaceutical (HMPAO and ECD are predominately cleared by the kidneys). Further discussion of radiation safety is beyond the scope of this article but are reviewed at length elsewhere (31–33).

**Figure 2:**
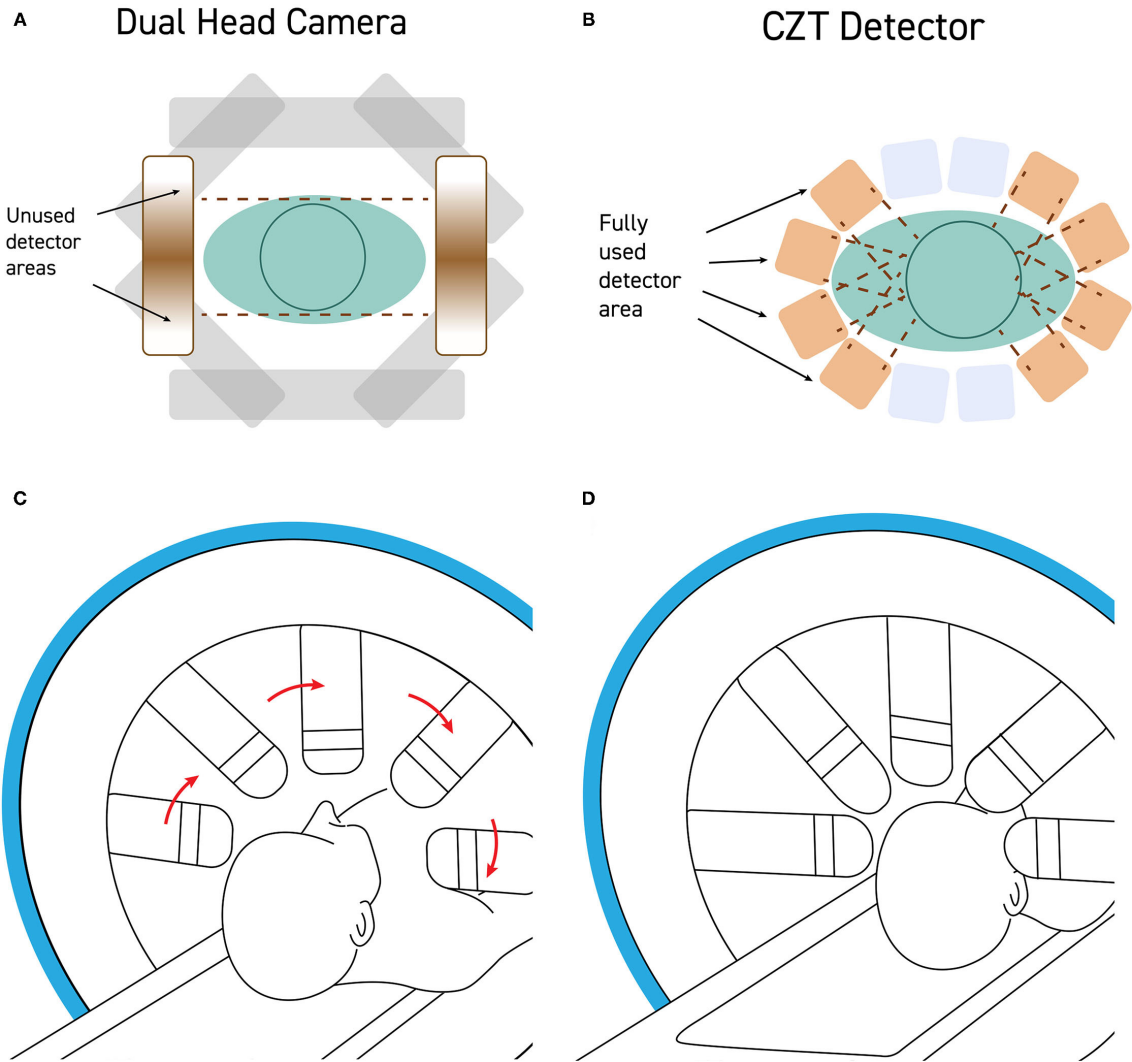
**(A)** Standard dual-head gamma cameras are too large to get close to the patient's head without hitting the shoulders during rotation. As a result, the field of view of the detector is often poorly utilized with a significant portion of the detector collecting no signal from the brain. **(B)** CZT detectors are quite small and can be mounted such that the detectors can get very close to the patient's head, maximizing the portion of the field of view occupied by the patient's head. **(C)** Modern CZT gamma cameras can be configured to withdraw the detectors heads during rotation and then **(D)** extend the detectors to close proximity of the patient's head during the scanning portion of a step-and-shoot protocol.

**Figure 3:**
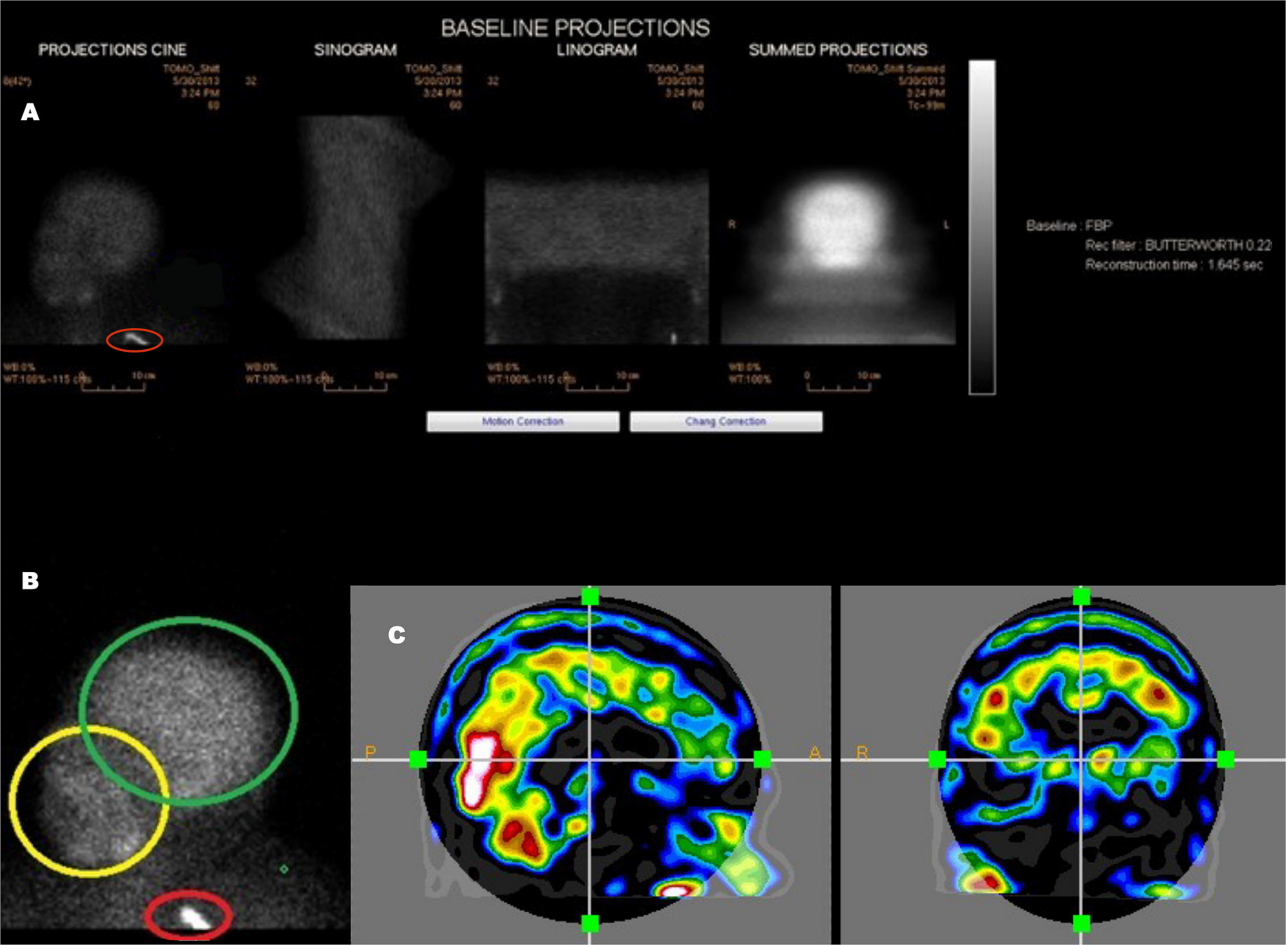
An example of a poorly executed perfusion SPECT scan. **(A)** Projection cine, sinogram, linogram, and summed projections illustrate minimal motion artifact. However, multiple technical errors are evident. The detectors were positioned excessively far from the patient and the acquisition zoom was not correctly set which resulted in large areas of empty space above the head and inclusion down to at least the clavicle in the field of view. In addition, a spill artifact is seen at the level of the clavicle (red circle). The area occupied by the organ of interest—the brain—makes up a small part of the acquisition matrix, best demonstrated in the summed projections. **(B)** A close-up view of the projection cine illustrated relatively high uptake in the facial region (yellow circle) relative to the uptake in the brain (green circle) and the spill artifact (red circle) contributing significant number of counts which are unrelated to the brain. **(C)** Sagittal and coronal alignment images illustrating considerable uptake in the salivary glands and free technetium in the scalp. All of these extraneous counts will reduce the statistics available from the brain. Furthermore, the low spatial resolution of this poorly executed scan is clearly evident.

Positioning the patient in the gamma camera is a critical step in the production of a quality SPECT scan (Figure 3). The camera heads should be as close to the patient as possible without actually hitting the head or shoulders during rotation. CTZ cameras will greatly alleviate the geometrical limitations posed by large sodium iodide detectors, as shown in Figure 2. The settings of distance from detector, acquisition zoom, and collection matrix determines the counts per pixel, as described below. Assuring that the correct matrix setting is utilized will maximize the statistics of the SPECT scan.

The patient's head should be positioned in the head holder in a comfortable manner. Soft padding and soft compression wrapping can be utilized to minimally restrain the head to assure the least motion. The patient should be instructed to avoid head movement and, in particular, avoid nodding the head. Rotational movement is exceedingly difficult to correct. Motion can be checked using the sinogram or linogram (see Figure 3) in the camera software. If motion is excessive, then the scan should be repeated. The consequences of poor technique and patient preparation are illustrated in Figure 2.

### Data Acquisition

As in all nuclear medicine studies a trade-off must be made between counts (statistics) and scan time. For patient comfort, shorter scan times are preferred, and it is recommended to keep acquisition time within the clinical tolerable limit (30 min). For good image quality, more counts are better, and for a brain SPECT scan it is recommended to acquire 10M brain-specific counts minimum. This will necessitate acquiring more than 10M total counts due to extraneous counts from non-neural structures (e.g., scalp, facial structures).

There are several factors that have an impact on the acquired number of counts in a scan. The following factors increase the number of counts, and they are reviewed below:

1. utilizing a camera with multiple detectors.
2. using a collimator with higher sensitivity.
3. increasing acquisition time.
4. increasing radiopharmaceutical dose.

#### Number of Detectors

Brain SPECT scans require a full 360° rotation around the subject's head. The total number of acquired counts increases proportionally with the number of detectors. Single detector cameras should not be utilized [as shown in Part I (2)]. The new CZT detector-based cameras will greatly enhance scan resolution and quality.

#### Collimation

The reconstructed resolution of a gamma camera system is determined largely by the collimator resolution. Brain SPECT perfusion studies are acquired with ^99m^Tc and require a “low energy” (LE) collimator. Collimator design is a trade-off between resolution, sensitivity, and septal penetration (rejection of unwanted photons). Because collimator terminology is not standardized between vendors, it is important to review the collimator specifications rather than relying on terms like high resolution (LEHR) or general purpose (LEAP).

#### Acquisition Time

The total number of counts in a SPECT scan is proportional to the acquisition time. Longer acquisition times will increase the susceptibility to patient motion which has a detrimental effect on image quality. Therefore, every effort should be made to maximize patient comfort during the scan. However, there is a limit to how far the acquisition time can be extended without risking patient motion. A practical limit is 30 min. For uncooperative patients, other measures may be necessary such as head restraints or sedation. Note that sedation can be used without interfering with the scan if it is administered after the injection of the radiopharmaceutical. Since the perfusion image is captured within ~40 secs of radiopharmaceutical injection and remains largely unchanged thereafter, tranquilizers or full sedation can be administered without altering the scan image.

#### Injected Dose

The total number of counts is proportional to the injected dose. It is recommended to maximize the allowable dosage without exceeding radiation limits for best results. Details are provided in the Canadian Association of Nuclear Medicine guidelines (30).

### Acquisition Matrix and Acquisition Zoom

The acquisition matrix determines the granularity of the acquired images. However, unlike in photography, a higher matrix in nuclear medicine does not necessarily produce a better image. The reason is that count statistics are low in nuclear medicine and pixel density drops by a factor of 4 for each doubling of matrix size. Lower pixel density means more noise which can be reduced by filtering, but filtering lowers the resolution of the image. Thus, there is a trade-off in choosing an optimum value for the matrix size in SPECT scans.

Typically, acquisition matrices are not arbitrary in size but limited to powers of 2. This reduces the useful choices for SPECT imaging to 3 values: 64, 128, and 256. The optimal choice is determined by the field of view of the detector and the expected/desired image resolution. It is the collimator, not the intrinsic resolution of the camera, that determines the overall resolution of the system. For a high-resolution collimator, the reconstructed resolution of a clinical SPECT scan using a sodium iodide crystal scintillation camera is on the order of 8 mm. The sampling theorem states that the sampling size (the pixel size) must be half, or less, of this value. This means the SPECT scan pixel size should be no larger than 4 mm. A general-purpose dual detector camera typically has a 20 in. field of view (FOV = 508 mm). Continuing with the example, to sample with 4.0 mm pixels, the required minimum matrix size would be 508/4 = 127. This means that in this example the 128 matrix is the best choice.

Thus far, it has been assumed the acquisition zoom is 1.0 (i.e., no zoom). Setting an acquisition zoom >1 means that the effective field of view of the detector is reduced by this factor. This is an option when the acquired object (the human brain in our case) is significantly smaller than the size of the detector. The zoom factor gives finer control over the pixel size as the increase/decrease of matrix size changes the pixel size by a factor of 2. For example, given a detector with a 15 in. field of view (381 mm), and the goal of a pixel size of 4.0 mm, the choice of matrix will affect the count density. The pixels size for a 128-matrix would be 381/128 = 2.98 mm, which is a bit smaller than needed. Count density is computed as the number of counts accumulated per unit area; a smaller pixel size therefore reduces the count density as there are more pixels per unit area. Since the area of one pixel is the square of its size, a reduction in size from 4.0 mm to 2.98 mm lowers the count density by a factor of 2. A 64-matrix size on the other hand gives a pixel size of 5.95 mm which is too large. However, the desired pixel size of 4.0 mm can be obtained by using an acquisition zoom of 5.95/4.0 = 1.49. This zoom will reduce the field of view by the same factor to 381/1.49 = 256 mm. If the patient's head is properly positioned this would just suffice to acquire a brain SPECT scan without truncation, while maximizing pixel count density and pixel size.

### Image Reconstruction

A series of raw images (i.e., projection data) as acquired by a SPECT camera cannot be interpreted directly but must first be reconstructed. Reconstruction is a part of the image generation process and should therefore be considered an extension of the gamma camera. In other modalities, such as CT, the user is often removed from the reconstruction process but in SPECT imaging, the choice of reconstruction parameters is paramount to the attainable image quality and depends strongly on the imaging equipment, the local acquisition parameters, and quite often the patient itself.

In terms of image reconstruction, a general distinction is made between filtered back projection and iterative reconstructions. They differ considerably and are discussed in some detail below.

There are other image processing functions that can be considered part of image reconstruction, most notably: attenuation correction, which is an essential element of brain SPECT imaging. Other optional functions include scatter correction and resolution recovery which are also discussed below.

#### Filtered Back Projection (FBP)

Back projection (BP) has traditionally been used, because it is simple, fast and readily available. While reviewed briefly here, a much more detailed description is provided in the Canadian Association of Nuclear Medicine guidelines (30). However, BP is not the best suited method for SPECT reconstruction. The prerequisites for BP to work correctly are that it is expected that the data has unlimited statistics and has perfect resolution (pencil beam reconstruction). For SPECT data these two requirements are not valid and represent an approximation. To deal with these limitations a filtering function is introduced to reduce the noise level in the reconstructed images to make them interpretable. The combination of filtering and back projection is commonly referred to as Filtered Back Projection (FBP). The filter is commonly implemented as a pre-filter, i.e., the raw projection images are filtered prior to back projection. The typical type of filter used in SPECT imaging is the Butterworth filter which is controlled by two parameters: cut-off and order. The order is usually fixed at 3 or 5, and the cut-off determines the final resolution and noise level (image texture). Please note that the cut-off is related to the sampling, i.e., the pixel size of the images. Higher sampling (i.e., smaller pixels) require a lower cut-off to achieve a similar smoothness compared to images acquired with larger pixels.

#### Iterative Reconstruction (IR)

Unlike FBP, iterative reconstruction is more of an umbrella term, which does not say much about the method or its performance. The Canadian Association of Nuclear Medicine guidelines (30) provides a more detailed discussion.

Two of the most common generic iterative reconstruction schemes are known as maximum likelihood estimation method (MLEM) and ordered subset expectation maximization (OSEM). The latter is more frequently used in SPECT imaging because it is a faster algorithm. For performance reasons, most OSEM implementations were initially in 2D (i.e., slice by slice) but nowadays most vendors have switched to a full 3D implementation. OSEM-3D is the preferred method in SPECT, because the limited resolution of the data results in considerable crosstalk between slices which is ignored in 2D implementations.

An iterative reconstruction engine goes through several iterations whereby the forward projection of the reconstructed slices is compared to the raw projection images. An error signal is added, or multiplied, to the synthetic projections and back projected again. This process repeats itself until the differences between the original projections and the computed projections are below a certain error threshold or until a set number of iterations is reached.

The most important advantage of iterative reconstruction in SPECT imaging is the fact that the imaging system (i.e., gamma camera and collimator) can be modeled in the algorithm, resulting in more accurate images. Iterative reconstruction methods tend to provide greater image contrast, i.e., the differences between areas of high and low uptake are enhanced and the overall dynamic range of useful information is extended. However, it also causes structures that appear singular and smooth in FBP to be visualized as clusters of hotspots. The images may appear sharper and of higher resolution, however the additional detail can appear noisy. A smoothing step is often utilized.

It should be noted that unconventional detection geometries such as line array detectors and multi-pinhole collimation, require specialized iterative reconstruction technique to produce images. However, the computational performance available today, allow such sophisticated algorithms to run in clinically acceptable time frames. In other words, these new technologies allow extraction of more information from scans performed under similar conditions.

### Attenuation Correction (AC)

All SPECT imaging is affected by attenuation which must be corrected for in brain imaging. The effect of attenuation depends on the energy of the emitted gamma quants, the density of the medium, and the distance traveled by the quant through the patient's body. The loss of transmission due to attenuation is an exponential function of distance. For ^99m^Tc (140 keV) the attenuation coefficient is 0.15 cm^−1^, which translates to a transmission loss of 50% when photons travel 4.58 cm through water (density = 1.0). As a result, photons originating from the center of the brain (basal ganglia) are detected with an apparent lower count rate than photons originating from the surface of the brain (cortex). Since the objective of brain SPECT perfusion imaging is to measure and compare regional blood flow in different functional areas of the brain; this cannot be done without attenuation correction.

Note: the theoretical attenuation coefficient of 0.15 cm^−1^ for ^99m^Tc in water must typically be reduced to 0.12 cm^−1^ to compensate for the presence of scatter. The exact value can be determined through a phantom measurement acquired under the same conditions as a brain SPECT scan.

The most common implementation of attenuation correction in brain SPECT imaging is a post-reconstruction technique based on the method developed by Chang (34). This method assumes that the attenuation within the patient's brain is uniform which is a first order approximation, because it does not consider the bony structures surrounding the brain. Given the overall resolution of SPECT imaging this simplification is acceptable because the differences compared to more accurate attenuation models are insignificant. Most modern cameras and software automate the attenuation process.

Hybrid SPECT/CT cameras have a CT scanner on board which can be used to obtain a real density map of the patient's head from which an attenuation map, a so-called μ-map, is computed. This μ-map is then used within the iterative reconstruction engine to correct for attenuation during the forward and backward projections, referred to as CT-guided attenuation correction (CTAC). Although the CT scan itself is of limited diagnostic use in brain SPECT perfusion imaging, and the CT scan adds to the total radiation exposure, it is still considered the most accurate implementation of attenuation correction.

### Resolution Recovery

The resolution of a gamma camera equipped with a collimator changes with distance. A parallel hole collimator basically is a slab of lead of a certain thickness, with lots of small (circular) holes in it. The intent is to only pass gamma photons that enter a hole perpendicular to the surface of the collimator. Photons arriving from different angles are attenuated by the lead walls between the holes and do not reach the detector. Due to the final length of the holes, they have an acceptance angle, i.e., photons arriving from angles that are slightly off perpendicular still make it to the detector. Looking back from the detector through the collimator holes, the circular area that is seen increases with distance which means the resolution of the imaging system decrease with distance. This is the reason why in nuclear medicine the imaging distance is so important to obtaining data of high quality (resolution).

The loss of resolution with distance is a pure geometrical effect and it is constant for a given collimator design. The collimator can be modeled in the iterative reconstruction engine with just a few parameters. During each forward and backward projection cycle the change in resolution is accounted for, and thereby resolves a higher resolution image. This method is also known as collimator deblurring which more accurately describes its function.

Today most iterative reconstruction implementations for SPECT imaging include this function. It is a good reason to switch from FBP to iterative as it brings a real advantage to the imaging chain.

### Scatter Correction

All gamma camera systems can acquire multiple energy windows simultaneously. This feature was originally developed for dual-isotope imaging to capture photon events at multiple energy levels. However, it can also be used to capture scattered events which can provide information that can be used to our advantage.

Scatter correction typically requires the acquisition of two additional energy windows, surrounding the photopeak window, in separate image channels (triple energy window technique). Because the scatter windows contain scattered events only, and their energy is in close proximation to the photopeak events, their noise spectrum is considered like the noise spectrum of the scattered events recorded in the photopeak window. By means of a weighted subtraction technique, the noise content of the photopeak images may be reduced; however, it can never be completely removed.

In theory, scatter correction will increase the signal-to-noise ratio of the acquired images. Phantom measurements are typically used to show its effectiveness; however, its performance on clinical data is highly dependent on the correct adjustment of weighting and care must be taken not to overcorrect the images. We want to separate the good counts (wanted signal) from the bad counts (noise), by subtracting out an estimation of the noise. Because the noise estimation can never be exact, the process can easily subtract too much and degrade our signal.

### Comparing Methods

**Figure 4:**
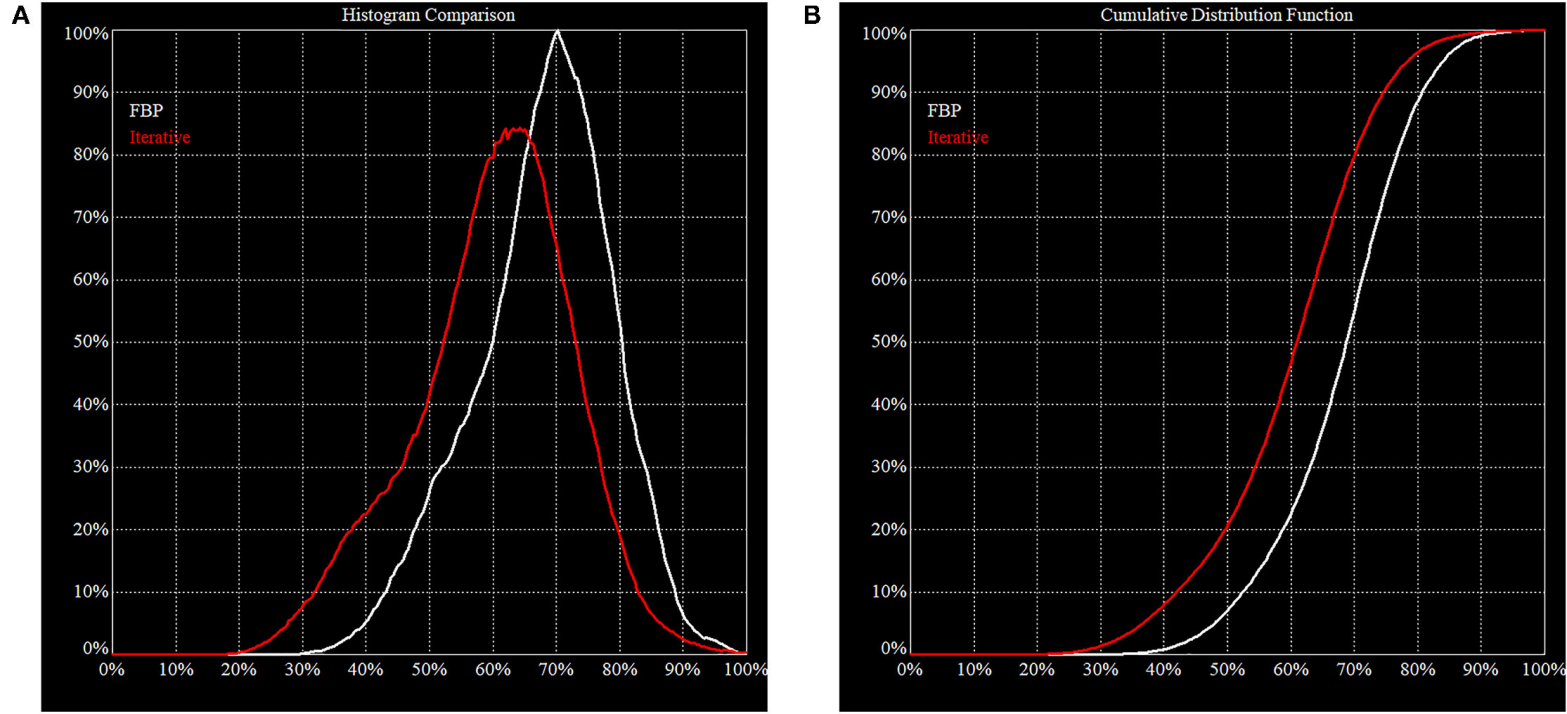
Histogram of FBP (white line) vs. Iterative Reconstruction (red line). These figures illustrate the difference in behavior of FBP vs. IR on the same clinical projection data. **(A)** The area under the histogram curves is the same but the peak is shifted downwards for IR. This is consistent with our expectation that IR will increase image contrast, i.e., the noise is pushed down, and a higher maximum is resolved. **(B)** In a cumulative distribution curve, the median count of the reconstructed volume is lower which must be considered when making direct comparisons.

Images produced by FBP and iterative reconstruction from the same projection data will be different (Figure 4). Most of the differences will be due to image texture (e.g., signal-to-noise ratio, resolution, etc). However, it cannot be excluded that a different image may lead to a different interpretation. This can be a complication, especially in a mixed environment. Because each brain is unique, it can take years of experience to become a fully-rounded reader. Therefore, it is so important to produce images in a consistent way. Despite their assumed superiority over FBP, the challenge for using iterative reconstruction is the lack of standardization. These issues are explored in greater detail in the Canadian Association of Nuclear Medicine guidelines (30).

### Reading a Perfusion SPECT Scan

The evaluation and interpretation of a brain SPECT scan is a complex matter, and each person is certain to develop an individual approach. Although there currently is no universal recipe for reading a brain SPECT scan, there are several basics that must be covered. Indications or disease states, including psychiatric indications were explored in detail in Part I of this two-part series (2) and are codified in the Canadian Association of Nuclear Medicine guidelines (30).

Before reading a brain SPECT scan, the reader should verify that the technical quality of the scan is acceptable. If the scan quality does not meet expectations, the patient should be re-scanned. In marginal situations, the reader should at least be aware of the technical problems and take them into consideration when interpreting the scan. The following list of items should be checked against the raw projection data:

(1) patient motion: re-scan the patient if too much motion.
(2) sufficient counts: >10 M are desired, <5 M unacceptable.
(3) absence of truncation: the entire brain should be in the field of view.
(4) sufficient delay between injection and imaging: minimal scalp and facial structure uptake.
(5) correct pixel size: 2–4 mm desired range.

Ideally, these items should be checked immediately after the scan and before the patient is released. The patient can be re-imaged within 2–3 h after injection without the need for re-injection.

Once the raw data is accepted, it can now be reconstructed. The checklist for the reconstructed data should include the following items:

(1) free from artifacts.
(2) properly masked: remove any activity outside of the brain, like salivary glands, nasal cavity, lacrimal glands.
(3) properly re-oriented.
(4) image resolution and texture correctly set (i.e., not too noisy, not too smooth).

SPECT scan data can be displayed in a number of ways. Tomograms provide the most anatomical information, particularly about deep structures. The tomograms and reconstructed images should be displayed in a suitable color scale. Although grayscale images are commonly used in radiology to visualize anatomy, it should be note that perfusion SPECT neuroimaging is a functional imaging modality, and most studies are best displayed in color as reviewed at length in Part I (2). To briefly reiterate, SPECT functional brain scans are demonstrating differences in perfusion as a one-off metric of neural activity. The difference in perfusion in an area of impaired function can differ by 12% or less and still have clinical significance. Several studies have shown that color vision is superior for detecting low contrast differences (35–38). While gray scale provides superior resolution of spatial details, it is considerably less sensitive at differentiating low-contrast signals. The selection of color scale is often a matter of personal preference; however, certain color scales have embedded reference to the physiological parameters of interest. In the case of perfusion SPECT neuroimaging, the use of a color scale that provide convenient and practical reference to the physiological properties of perfusion is highly recommended.

The regional cortical counts of both ^99m^TC-HMPAO (39, 40) and ^99m^TC-ECD (41) have been compared to direct measurements of regional cerebral blood flow using ^133^Xenon. Both neurological and psychiatrically normal subjects and subjects with known perfusion defects were scanned both with ^133^Xenon and the respective radiopharmaceutical sequentially in the same scanner with no change in position. Regional cerebral blood flow (rCBF) was calculated by the method of Kanno and Lassen (42) from ^133^Xenon data. Linear regression analysis was utilized to characterize the relationship between count densities for ECD or HMPAO and rCBF based on ^133^Xenon. For ECD, the correlation was good (r = 0.88) with a slope of 0.83 (41). In the case of HMPAO, the correlation was also good (0.92) with a slope of 0.82 (40). Both ECD and HMPAO correlate well with ^133^Xenon studies over physiological ranges but do underestimate blood flow at high velocities (40).

**Figure 5:**
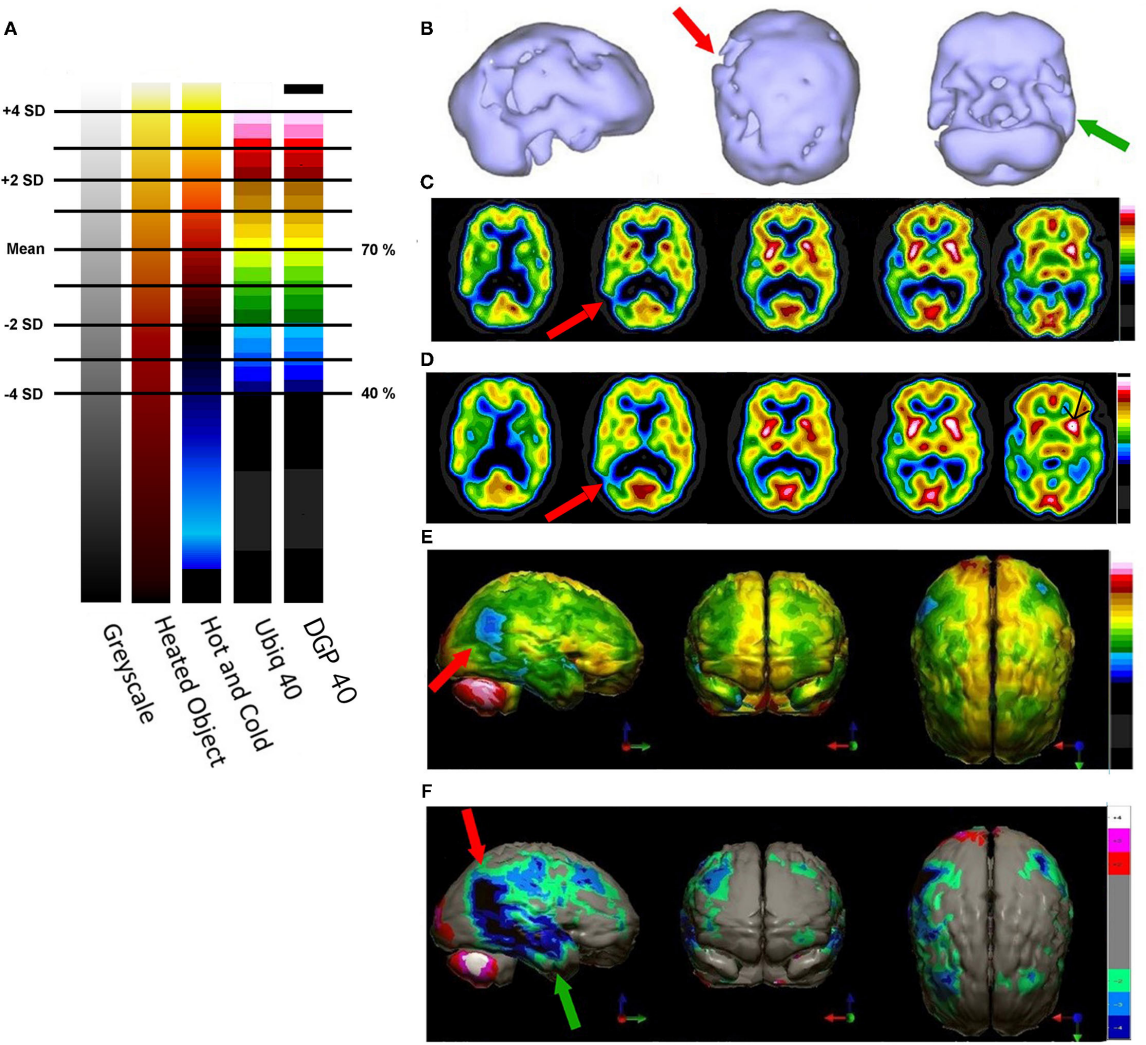
**(A)** Various commonly used color scales and a greyscale are displayed. The mean cerebral perfusion in the human brain is 70% of the maximal flow with a standard deviation (SD) of 8.35%. The mean and ± 1 and 2 SD are indicated. A change of ± 2 SD is unlikely to be appreciated in greyscale but can be readily distinguished in Heated Object and Ubiq40 color scales. An increase of 2 SD can be distinguished in Hot and Cold color scale, but a decrease of 2SD or less would not be discernable. A 1 SD increase or decrease would be difficult to discern in greyscale, Heated Object and Hot and Cold color scale, but are readily detected in Ubiq40 and DPG40. **(B)** A 58-yr-old female was struck on the right parietal region by a heavy object with loss of consciousness of ~2 h. Perfusion SPECT scan was performed 7 years after the injury with ^99m^Tc-HMPAO and a dual-head gamma camera. SPECT data can be displayed in 3-D representations that facilitate the identification of large, diffuse, or subtle lesions. Here, data is presented as an isocontour display wherein cortical areas which fall below 60% of the maximal cerebral blood flow are displayed as a depression or hole. The large parietal defect is apparent on the right (red arrow), as well as bilateral temporal lobe hypoperfusion (green arrow). **(C)** 4 mm horizontal sections illustrate decreased perfusion in the right parietal region (red arrow). The color scale is the Ubiq40. **(D)** 4 mm horizontal sections illustrate decreased perfusion in the right parietal region (red arrow). The color scale is the DPG40. Note the black spot at the point of highest perfusion in the left thalamus (black arrow). **(E)** 3-D representation utilizes the Ubiq40 color scale. The right parietal defect appears as an area of blue and green (red arrow). **(F)** The patient's data is compared to a normative database (*N* = 68). A map of statistically significant differences can be generated using the Oasis software by Segami, Inc. Here, the color scale indicates gray for areas that do not differ significantly from the normative database. In contrast, areas of green, light blue, and dark blue represent areas of more than 2, 3, and 4 SD below the mean perfusion of the normative database, respectively. Statistically significant increases in perfusion are illustrated in the red color scale. The parietal lobe injury (red arrow) and the contra-coup injury are easily visualized, along with more diffuse penumbra injury and bilateral lateral temporal lobe hypoperfusion (green arrows).

The same group (43) had earlier examined ^133^Xenon CBF in 97 volunteers free of psychiatric or neurological conditions. They determined that the mean CBF was 71 ± 12 ml/min/100 g of grey matter. A number of groups have examined CBF in whole brain, gray matter and white matter using Xenon SPECT (43–47), Xenon CT (48, 49), ^15^O-water PET 48, and arterial spin labeling MRI (50–52). Across the techniques and research groups, the estimated mean CBF in gray matter is 70.3 ± 8.35. Figure 5 illustrates the color scales with the mean and standard deviations labeled. In addition, a case is illustrated using isocontour representation, two different, but similar color scales (described below) and a statistical comparison to a normative database. The figure is an adaptation of Figure 4 from Part I of this series (2) and the case is more fully described therein.

For brain SPECT perfusion studies, we recommend the DGP40% scale for HMPAO (illustrated as the color scale on the right in Figure 5) and the DGP35% scale for ECD studies. A very similar, earlier, rendition of these color scales is the Ubiq40, also illustrated here. These color scales were specifically developed for brain SPECT imaging by one of the authors (DGP) and they have several design features that make them especially useful. These two scales are static and do not require any manual window leveling; in fact, the user should refrain from making any window level adjustments to allow for consistent and reproducible settings. The two scales have a lower threshold of 40 and 35% respectively, removing any background noise that would otherwise interfere with the images. These color scales are discrete and are made up of 21 color bands of a fixed width. The color progresses in a natural fashion from dark blue to white, based on the geographic model. The very top of the scale is black, which allow the reader to immediately locate the hottest area in the scan as it appears as a small black spot or area within a larger white area. The location of the maximum is an important clinical parameter. Typically, the maximum is located in the cerebellum (HMPAO) or visual cortex (ECD). A maximum located elsewhere (e.g., thalamus) is an important clinical finding.

The SPECT scan images should be read in a systematic fashion. In one approach, the 3-D reconstructions are examined to detect any large or subtle areas of hypoperfusion or increased perfusion which might be missed in the tomograms. Cortex wide patterns, such as diffuse scattered hypoperfusion will be evident. Alternatively, the tomograms are initially evaluated. Regardless, each area of the brain should be consciously visualized.

**Figure 6:**
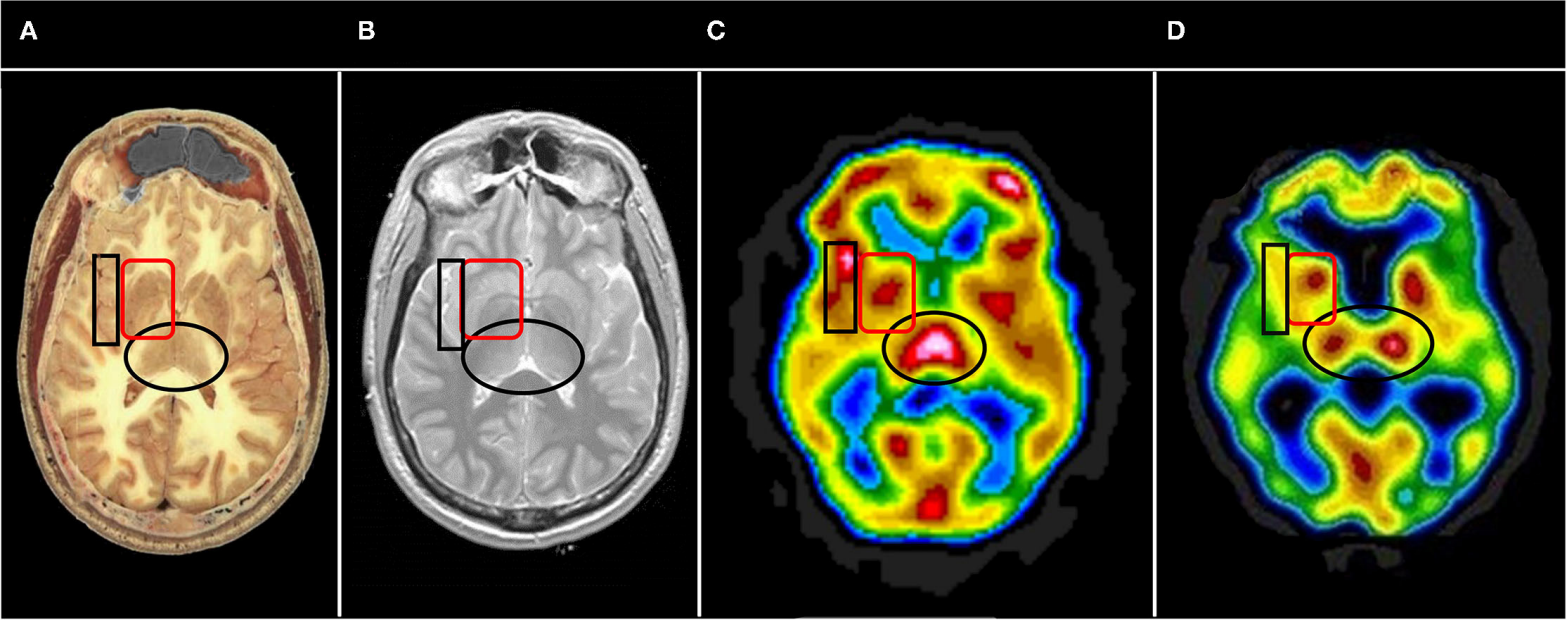
Representative horizontal sections at the level of the thalamus and basal ganglia from **(A)** human cadaver, **(B)** MRI, **(C)** SPECT of healthy individual, and **(D)** SPECT of individual with advanced Alzheimer's disease. The thalami are indicated by the black oval in each frame. In healthy individuals the thalami are closely approximated with a narrow 3^rd^ ventricle separating each side. Note the separation of the thalami in **(D)**, consistent with volume loss due to degenerative changes. The basal ganglia are indicated by the red rounded square. The insula is indicated by the black box. Note the close approximation of the insular cortex to the lateral aspect of the basal ganglia separated only by the thin external capsule.

Often, it is useful to begin with the horizontal tomograms. Careful attention should be given to the subcortical structures, such as the thalami, basal ganglia, cinguli, and the brainstem (see Figure 6). Then attention should be turned to examine each area of the cerebral cortices, including the insula, bilaterally. The cerebellum should be examined with attention to the vermis and the hemispheres. Structures should be examined in all three planes (horizontal, coronal, sagittal) as abnormalities may be apparent in one plane but hidden in other planes. Areas of overactivity and areas of decreased activity should be noted. Asymmetry should be noted. The pattern of perfusion should be noted in the 3-D reconstructions.

Particular attention should be given to the posterior cingulate in cases of suspected dementia, given the high sensitivity and specificity of this structure in the diagnosis of Alzheimer's disease (2). The basal ganglia and anterior cingulate have particular relevance to OCD. The orbitofrontal cortex should be examined both in tomograms and in 3-D reconstructions if executive dysfunction (ADHD, TBI etc.) are being considered (30). Understand that multiple findings are the norm, rather than the exception, as comorbidity is common. If an area of hypoperfusion is detected, then the size and structures involved should be noted to facilitate clinical correlation. For example, if hypoperfusion is seen in the inferior occipital cortex and inferior parietal cortex on one side (suggestive of posterior cerebral artery infarct), then involvement of the thalamus and basal ganglia, as well as changes in the contralateral cerebellum, should be assessed. While it is important to look for a primary cause of the patient's symptoms, it is vital to remember that most patients have co-morbidities (see Part I), and these co-morbidities play a pivotal role in effective treatment planning. A full discussion of the reading of a SPECT scan is beyond the scope of this paper and will be addressed in the future.

### Statistical Analysis

#### The Normal Database

Statistical comparison to a normative database is often cited as an important step in the characterization of a pathological SPECT scan. However, the definition and vetting of a normative database is complicated. The first issue is that each radiopharmaceutical has a different activity distribution and vulnerability to back diffusion as detailed in Part I of this two-part series (2). For example, ECD favors the temporal lobes, while HMPAO levels tend to be lower there. Technically, each radiopharmaceutical should have its own normal database. This is not surprising as we would not expect to use the same normal database for FDG-PET as we would for HMPAO. The second issue is how is a “normal” subject defined. Ideally, the subject should be free of psychiatric illness or symptoms, free of alcohol, tobacco, marijuana, or illicit drug use, without family history of first° relatives with psychiatric or neurological illness, and without history of concussion or TBI. Structured clinical interviews, extensive questionnaires, and drug screens can help to obtain a population free of exclusion criteria (53–55). To quote Dr. Mena's seminal work:

“*The procedure for the selection of the normal pediatric subjects was as follows: the children were selected from two sources: a) those attending elective non cranial surgery at a public hospital and b) volunteer health personnel relatives. Neuropsychiatric screening included: a) semi-structured interview of the mother and child, b) mental status and neurologic examination performed by two child psychiatrists. Exclusion criteria included the following: positive pre-, peri- or post-natal history, developmental disorder or delay of any kind, learning disorder, psychiatric disorder or isolated emotional or behavioral symptoms, severe family psychopathology or neurologic disorder, abnormal neurologic examination, and school underachievement. Using these criteria more than 50% of potential subjects were rejected.”* (53).

Dr. Amen has taken a similar transparent and rigorous approach to defining a normal database:

*The Control group was recruited using local advertisements in newspapers and local colleges. Each subject met the clinical criteria for a healthy brain subject based on our criteria that included the absence of current medical illnesses, brain trauma, family history of psychiatric illness, drug/alcohol abuse and no current or past evidence of behavioral or psychiatric issues as measured by a detailed clinical history, Minnesota Multiphasic Personality Inventory (MMPI) and Structured Clinical Interview for Diagnosis (SCID) for DSM-IV* (55).

Both male and female subjects should be included as there are perfusion differences between the genders (56). Also, a wide age range should be included in the database. Perfusion changes with age (54, 57). Most statistical analysis programs separate the normal database into age groups. The index patient's data is compared to the appropriate age-specific subgroup.

#### Statistical Analysis of SPECT Scan Data

The statistical analysis of perfusion SPECT scan data can be conducted at a number of levels. The simplest approach is a regions of interest analysis. Early studies defined regions of interest visually and compared data within these regions to identify differences.

A more rigorous statistical analysis of brain SPECT data can be either voxel-based or region-based; however, in both cases it is necessary to spatially normalize the data first. Examples of such spatial normalization are the Tailarach atlas (58) and the Montreal Neurological Institute atlas (59). These methods are based on a registration of anatomical references which are extracted by the software algorithm from the image data. The registration is non-linear (i.e., requires warping), because human brain shapes differ substantially between subjects. As a result, the temporal pole of the index patient can be mapped precisely to the temporal pole of the comparator group or normal database. This process is certainly not unique to the analysis of perfusion SPECT scans and is widely used in both functional and anatomical neuroimaging. The advantage of analyzing individual voxels is that it tends to be more sensitive; however, the disadvantage is that the result may not be meaningful to the reader/clinician, because of the limited context. Regions on the other hand, are logical groups of voxels that are based on anatomical or functional areas (e.g., Brodmann areas) in the brain which are more relevant to the reader/clinician. For example, data within regions of interest can be compared statistically across conditions or against a control dataset (20, 21, 55).

In addition to a spatial normalization, the SPECT scan data also must be normalized in intensity because the measured blood flow by either ECD or HMPAO is relative. HMPAO and ECD trapping is not perfectly proportional to rCBF as measured by ^133^Xenon studies (60, 61). While generally these pharmaceuticals are rapidly converted to a hydrophilic form and become trapped (as described in Part I), the trapping is not perfect, nor instantaneous (A more significant redistribution occurs with ^123^I-IMP as described in Part I). As a result, there is not an exact linear proportionality between rCBF and retention of ECD or HMPAO. Nonetheless, the distribution and level of radiopharmaceutical closely approximates rCBF over physiological ranges (60).

Typically, the images are normalized to the maximum value in the subject's brain—this is most often the cerebellum or the visual cortex. Although the maximum value is a somewhat noisy parameter, it actually works quite well in SPECT imaging due to its somewhat limited spatial resolution and the smoothing of the data as part of the reconstruction process, as described above. Sometimes, the maximum value in the subject's brain is elsewhere, such as the thalamus. The biasing of the data that can result in this situation is avoided by using the cerebellum or visual cortex consistently; however, there is a caveat. In the situation in which the cerebellum is damaged or there is cross-cerebellar diaschisis, then a cerebellar normalization can falsely elevate the findings in the remainder of the brain. Hence, attention to each brain scan is necessary to avoid these errors.

At this point, a statistical analysis can be performed on the index patient's data. For each pixel, a comparison is made to the range of pixel values at the same spatial point within the reference dataset. The resulting data can be displayed as charts, graphs, or as 3-dimensional surface displays (62–65). A color-coded map of statistically significant differences can be generated as illustrated in Figures 2–6, **9** in Part I of this two-part series (2), as well as in Figures 5, **10**, **12** below using software from Segami Corp. (Columbia, MD). Statistical parametric mapping has been used to differentiate AD from controls with high accuracy (3, 66–69).

#### Artificial Intelligence and Machine Learning

Artificial Intelligence (AI) and machine learning are currently gaining interest in the research communities of almost all fields because it can be performed at low cost and with potentially high benefits. However, the application in clinical brain SPECT imaging has not been established. In general, these methods rely on huge quantities of data and are an attempt to automate the comparison process, but also allow artificial intelligence algorithms to explore the data in novel and/or complex ways. These methods attract considerable attention, but the reader and reviewer must be careful to distinguish research applications and clinical applications, as well as statistically significant differences which lack clinical meaning from those with substantial clinical significance. Nonetheless, as AI has become more sophisticated, it is possible for a program to parse the data in novel and unexpected ways as the program learns from the data.

Because AI can apply complex calculations to datasets iteratively and at high speed, it is possible to test the data against itself. The software can repeatedly select subsets of data to serve as a temporary reference or rule dataset and then iteratively compare the remaining data against this rule dataset. By repeating this process over and over with different temporary rule datasets, a new feature can be identified, or an identified feature can be confirmed as valid. While unsupervised learning by AI algorithms may yield clinically irrelevant features, both supervised and unsupervised learning algorithms may produce or verify clinically recognized features, known from decades of visual reads of SPECT scan data. AI techniques have been applied to attenuation correction with promising results (70–72). Improved classification of ^123^I-ioflupane (DatScan) findings for differentiating Parkinson's disease from other neurodegenerative disorders has been demonstrated (73, 74). Perhaps the most interesting recent development is the first steps in creating an AI driven SPECT image reconstruction algorithm (75).

Several groups have explored machine learning algorithms to differentiate Alzheimer's disease from controls. The process of developing a pattern-recognition algorithm for distinguishing a disease state from controls or another disease state begins with creating a training data set. Carefully selected cases of the disease state (e.g, AD) are collected. The size of this training data set must be large enough to allow a robust signal (e.g., decreased parietal and posterior cingulate perfusion) against the background noise of intersubject variability. Then algorithms can be applied to separate the data into different categories. Support vector analysis (76), which identifies multiple features that distinguish one group from another, is one form of analysis and it has been used to differentiate AD from controls yielded a sensitivity of 97%, a specificity of 100%, and an accuracy of 99% (77). Principal component analysis is another method which extracts features by representing the data in a covariance matrix (78, 79). The algorithm can then be trained on the training set by using a series of subsets to compare back to the training data set. The training data set is randomly divided into a number of subsets. Then N-1 subsets are then tested against the left-out subset. This process is repeated sequentially leaving out a different subset to be used as the test subset. After thousands of iterations of this process, the best classification rule can be determined. The method is often referred to as “leave one out cross-validation.” Finally, the machine learned algorithm can be compared to a new set of data to validate the accuracy of the process in differentiating one group (disease state) from another (control or different disease state).

## Combining Perfusion SPECT Scans With Other Modalities for Improved Diagnostics

The authors realize that perfusion SPECT neuroimaging, while extremely sensitive, needs to be used in the context of complete patient clinical information - history, physical examination, other imaging and laboratory tests, and other neuropsychiatric evaluations to be of greatest value. Perfusion SPECT neuroimaging adds valuable neurobiological information to the subjective realm of symptomatology. Furthermore, perfusion SPECT can add additional dimensions to the results of other neuroimaging modalities resulting in better and more reliable differential diagnoses. We will illustrate this point with four situations commonly encountered in psychiatry and neurology.

### Situation 1

The first scenario is a 72-year-old patient with a 2-year history of progressively worsening memory problems. She denies hallucinations, tremor, or difficulty with her gait. The long-time course makes delirium less of a consideration. The absence of Parkinsonian symptoms reduces the need to consider that group of disorders. The patient undergoes a Montreal Cognitive Assessment (MOCA) and scores 22/30 placing her in the mild cognitive impairment (MCI) range. However, is this early AD, early vascular dementia, early FTD, or early Lewy Body dementia (LBD) without tremor? An MRI might show widening of the sulci, but this might be no greater than is expected for age. The neurologist might, at this point, order an amyloid scan. If the amyloid scan is positive, then we can proceed on the assumption that the patient has MCI of the AD type; however, there is a false positive rate among aged normals which is due to increasing nonspecific binding with age. Approximately 20% of controls at age 60 years and 40% of controls at age 80 years had false-positive scans (80, 81). Thus, there is a >20–30% chance that the patient does not have AD, even with a positive amyloid scan. The situation is even more dire if the amyloid scan is negative. We can rule out AD as the cause of the patient's memory problems, but we cannot narrow the differential any further.

Unfortunately, the amyloid scan is a binomial test—the result is either positive for AD or negative. The amyloid scan can yield no further clues in the differential diagnosis. This is where perfusion SPECT or FDG-PET can be highly beneficial (3, 82). By following up with a perfusion SPECT scan, the diagnosis may be revealed. For example, if there is frontal and temporal lobe hypoperfusion with a negative amyloid scan, the likelihood of FTD is greatly increased. An example of fronto-temporal MCI is illustrated in Figure 2 of Part I of this two-part series (2). If there is hypoperfusion of the occipital lobes, then the risk of LBD increases substantially, even in the absence of tremor (83) (A follow-up DaTscan might be indicated at that point). If diffuse hypoperfusion is found, then a number of differential diagnoses need to be considered (82, 84), including toxic brain injury, diffuse post-concussive brain injury, vascular dementia, and infectious brain injury. The research literature supporting SPECT findings in neurotoxicity were extensively reviewed in Part I of this series (2). A case of infectious brain injury is illustrated below.

Recent work on PET markers for tau (e.g., AVI451, also known as ^18^F-flortaucipir and the tradename Tauvid^TM^) have been fruitful and extensive literature now exists on tau protein labeling in AD (85–88). In contrast, the tau imaging characteristics of chronic traumatic encephalopathy (CTE) remain poorly understood with a dearth of studies (89–91). Pathological studies have confirmed that CTE (92) is characterized by a distinctive accumulation of tau and neurofibrillary tangles in a perivascular distribution in an irregular pattern in the cortex favoring the depths of cortical sulci (93–95). Furthermore, the tau protein tends to predominate in cortical layers 2 and 3 (96). This is distinct from the pattern seen in AD (88, 96, 97) wherein tau accumulates in cortical layers 3–5 involving both sulci and gyri and with a preferential accumulation in the precuneus, posterior cingulate gyrus, hippocampus and subiculum (88, 98–100). It also stands in contrast to the findings in progressive supranuclear palsy (PSP) wherein the accumulation of tau protein is distinctive in the cerebellum and cerebellar dentate nucleus (101, 102) or in corticobasal degeneration (CBD) wherein tau accumulation is found in the striatum and globus pallidus (although severe CTE can show subcortical accumulation of tau, but this tends to be in the mamillary bodies, thalamus, and other structures vs. the basal ganglia) (95, 103). A recent small study utilized ^18^F-flortaucipir to predict amyloid status (regardless of diagnosis) vs. controls with a sensitivity of 94% and specificity of 83% (104). However, ^18^F-flortaucipir binding in early AD (Braak stages I-IV) is much less reliable and likely will hamper its efficacy in predicting MCI type and progression (88). At least one longitudinal study has shown a small predictive value (risk ratio 1.40) for ^18^F-flortaucipir scan in the progression mild cognitive impairment (105). The utility of tau neuroimaging as a predictor in preclinical and prodromal stages of AD remains uncertain (106). Moreover, the presence of tau binding in mild TBI (107), FTD, PSP, and several other degenerative disorders clouds the picture further.

As detailed in Part I of this two-part series (2), perfusion SPECT neuroimaging has high sensitivity (89%) and specificity (89%) for differentiating AD from controls. When paired with quantitative analysis, perfusion SPECT can predict the progression of MCI to AD with a sensitivity of 97% and a specificity 100%. FTD can be distinguished from AD with a sensitivity of 96% and a specificity of 80% (2, 3, 30). Perfusion SPECT scans can clarify the differential diagnosis in cases of cognitive impairment, MCI, and dementia.

### Situation 2

The second scenario involves a 54-year-old patient early in the progression of Parkinsonian symptoms. Tremor, postural instability, and bradykinesia might predominate. But what is the diagnosis? Statistically speaking, there is a fair chance it is Idiopathic Parkinson's disease (IPD). However, multiple other Parkinsonian syndromes cannot be ruled out.

IPD is a progressive neurological disorder characterized by selective degeneration of dopaminergic neurons in the substantia nigra. While IPD may be the most prevalent form of parkinsonism, it shares, in part, symptoms with progressive supranuclear palsy (PSP), multiple system atrophy (MSA), corticobasal degeneration (CBD), vascular parkinsonism, and dementia with Lewy bodies (DLB). Symptoms of tremor, bradykinesia, postural instability, rigidity, and autonomic dysfunction can overlap, to a greater or lesser degree, in each of these disorders. When only tremor is present, differentiation from essential tremor also is relevant. Specific clinical symptoms, such as cerebellar signs in MSA, gaze palsy in PSP, atrophy of the mesencephalon seen with MRI in PSP, or overt dementia and visual hallucinations in DLB, may only manifest at a later stage of the disease. Given the multifarious presentation of IPD, the discrimination of these diseases ***early in the course of illness*** can be challenging. Yet, the correct diagnosis is critical, as each disease process has a different pathology, different progression, and different response to medication. No single test has emerged as capable of sufficient sensitivity and specificity to serve as a stand-alone diagnostic tool.

Dopamine transporter proteins (DAT) can be visualized in the striatum using ^123^I-ioflupane or ^123^I-FP-CIT, commercially marketed as DaTscan (108). ^123^I-ioflupane demonstrates decreased DAT density in the striatum and caudate nucleus consistently in IPD. The challenge has been to differentiate IPD from other parkinsonian syndromes. For example, MSA cannot be reliably distinguished from IPD based on DAT labeling alone (109). However, the combination of decreased striatal DAT labeling and increased striatal perfusion (metabolism) appears to be highly sensitive and specific for IPD, differentiating IPD from MSA in up to 99% of cases (110). By way of contrast, the Unified Parkinsons' disease rating scale (UPDRS) can readily differentiate IPD or MSA from controls with a sensitivity of 91%, but the UPDRS cannot distinguish IPD and MSA (111, 112).

**Figure 7:**
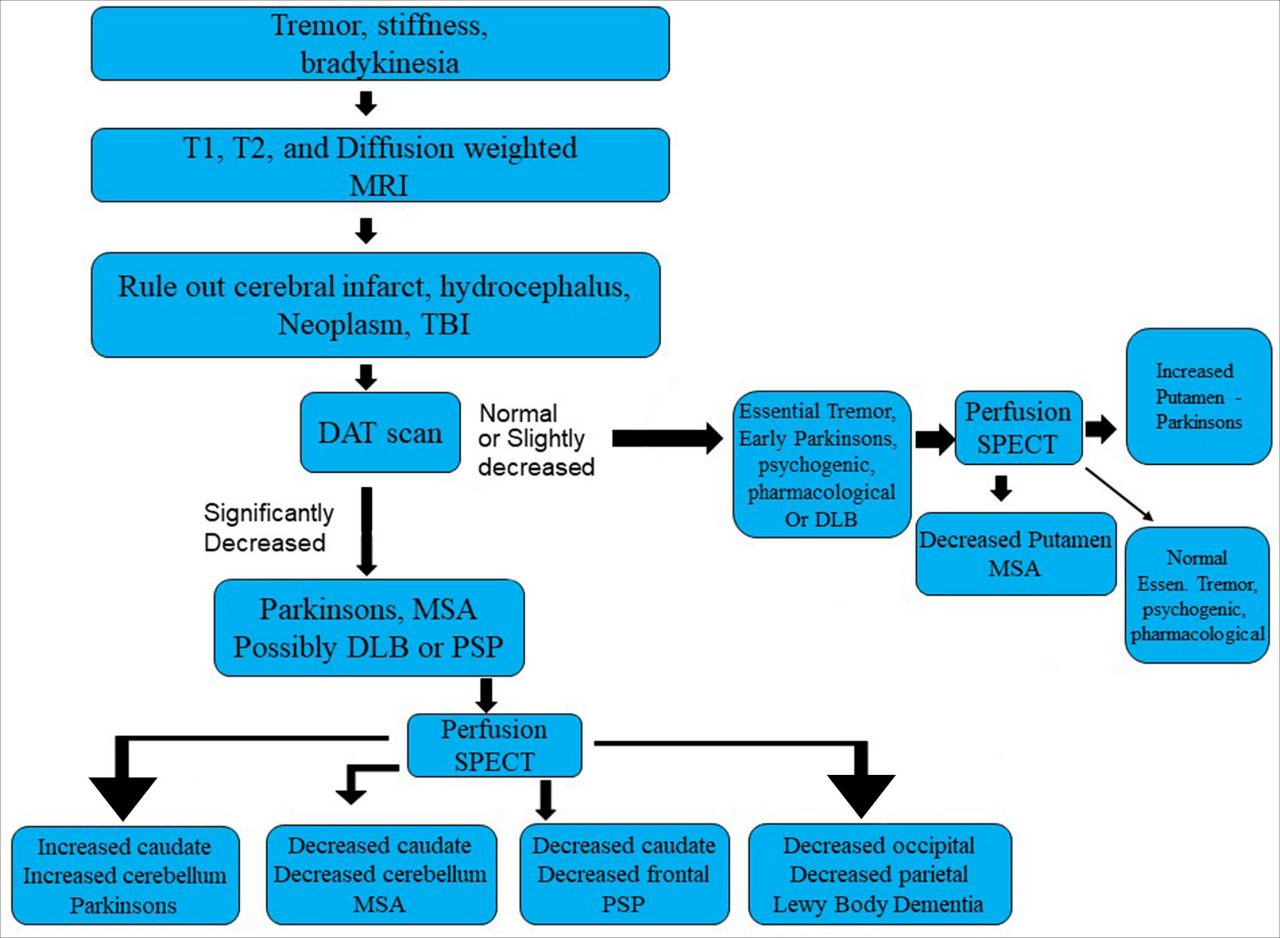
Flowchart for evaluation of Parkinsonian syndromes.

SPECT perfusion neuroimaging repeatedly has shown an increased perfusion of the striatum in IPD (112–116). In contrast, hypoperfusion (or hypometabolism) in the striatum is consistently reported in MSA using SPECT or FDG-PET (110, 115, 117–119). Similarly, perfusion tends to be increased in the cerebellum in IPD (112, 115, 116, 120–122), while cerebellar perfusion (or metabolism) is decreased in MSA (113, 123). For example, Van Laere and colleagues (110) utilized ^99m^Tc-ECD and ^125^I-FP-CIT to study perfusion and DaT binding in patients with IPD, MCA, PSP, and LBD. Patients with MSA showed a statistically significant decrease in perfusion in the bilateral posterior putamen and cerebellar vermis and hemispheres relative to patients with either IPD or essential tremor. Patients with PSP demonstrated decreased perfusion in multiple areas, including left frontal lobe, left caudate, anterior cingulate, and thalamus relative to IPD. Patients with DLB were distinguished by pronounced hypoperfusion of the posterior temporoparietal cortex bilaterally. Patients with IPD demonstrated significantly increased perfusion of the cerebellum relative to essential tremor, MSA, PSP, and DLB (124). Notably, DaT binding alone did not consistently distinguish among the different diseases with an accuracy of only 58%. However, the combination of the two techniques increased classification accuracy or differentiation of the degenerative diseases to 99% (110). See Table 1 and Figure 7 for a diagnostic flow diagram. These data support the increased use of perfusion SPECT, in combination with DaTscan, anatomical MRI, and other tools, in the differential diagnosis of Parkinsonian syndromes.

**Table 1:** The role of functional neuroimaging in the differential diagnosis of Parkinsonian syndromes.

	**Essential tremor**	**Idiopathic Parkinson's disease**	**Multiple system atrophy**	**Progressive supranuclear palsy**	**Lewy body dementia**
Symptoms	Tremor	Tremor	Tremor	Tremor	Tremor
		Bradykinesia	Bradykinesia	Bradykinesia	Bradykinesia
		Rigidity	Rigidity	Rigidity	Rigidity
		Autonomic Instability	Autonomic Instability	Autonomic Instability	Autonomic Instability Late Visual Hall.
Course	Stable	Progressive	Progressive	Progressive	Progressive
DAT	DAT—normal	DAT—decreased Statistically Significant	DAT- decreased Statistically Significant	DAT—decreased variable	DAT—slightly decreased
Perfusion SPECT or FDG PET	Normal	Increased in putamen Increased in cerebellum Decreased parietal lobes	Decreased in putamen Decreased in cerebellum	Decreased left frontal Decreased left caudate Decreased thalamus Decreased ant. cingulate	Decreased in occipital Decreased in parietal
UPSIT	Normal	Impaired	Normal	Variably impaired	Impaired

### Situation 3

**Figure 8:**
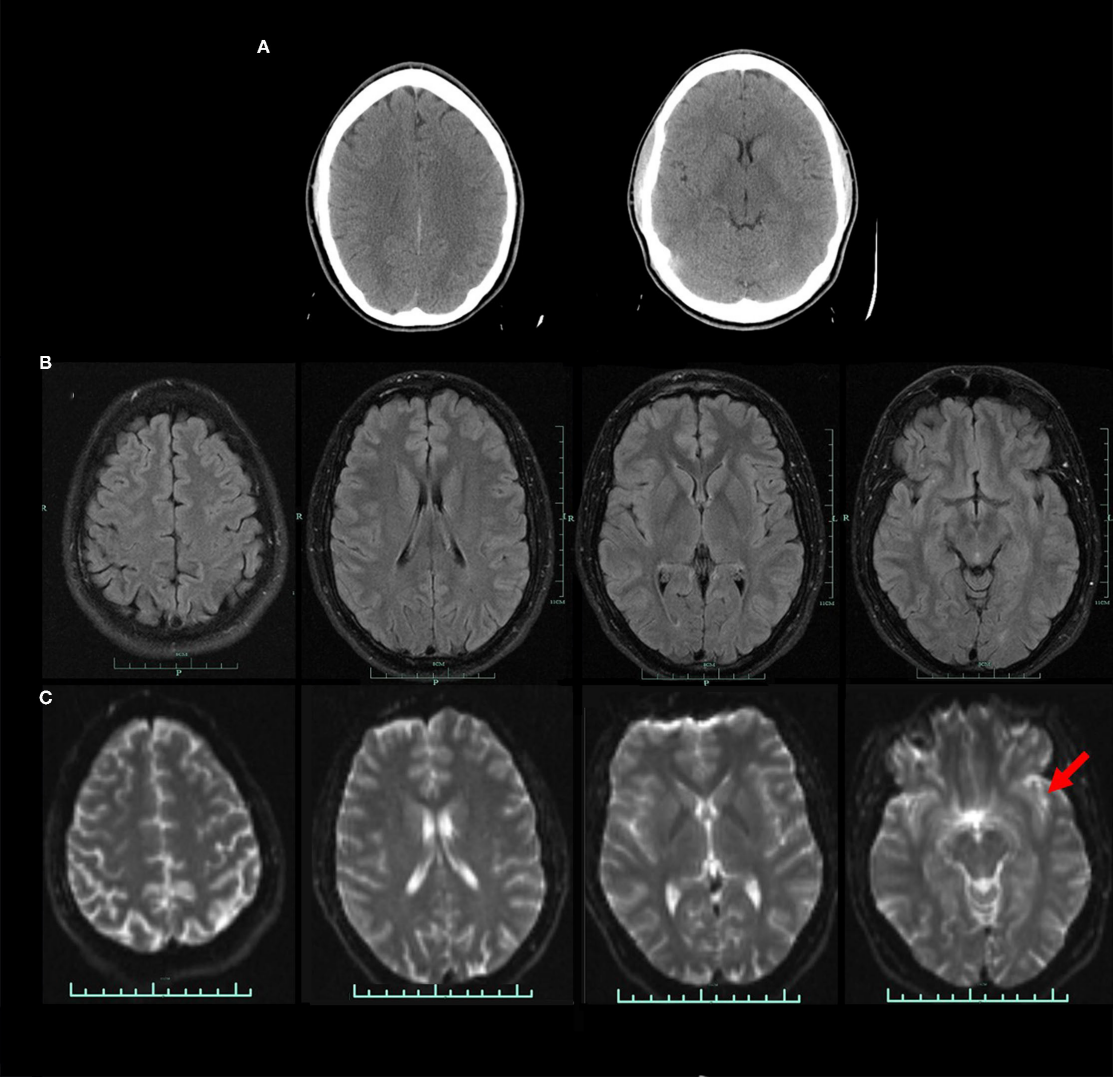
**(A)** CT scan at two levels obtained in the acute setting. CT read as normal. **(B)** FLAIR protocol MRI at four levels. No abnormality visible. **(C)** DTI MRI at four levels. A small area of high signal is noted in the left temporal lobe (red arrow).

The third scenario involves a 29-year-old male who was involved in a motor vehicle accident. The patient was stunned at the scene but there was no evidence of loss of consciousness. He was taken to the emergency room and underwent a CT scan, which was read as normal (Figure 8). Within days of the accident, the patient was experiencing severe headaches, photophobia, and memory problems. Nine months later, the patient had persistent symptoms of frequent headaches, memory difficulties, cognitive slowing, and irritability. The patient underwent an anatomical MRI. This showed a slight high signal abnormality in the left temporal lobe visible only on T-2 weighted images (Figure 8). He then underwent a perfusion SPECT scan.

**Figure 9:**
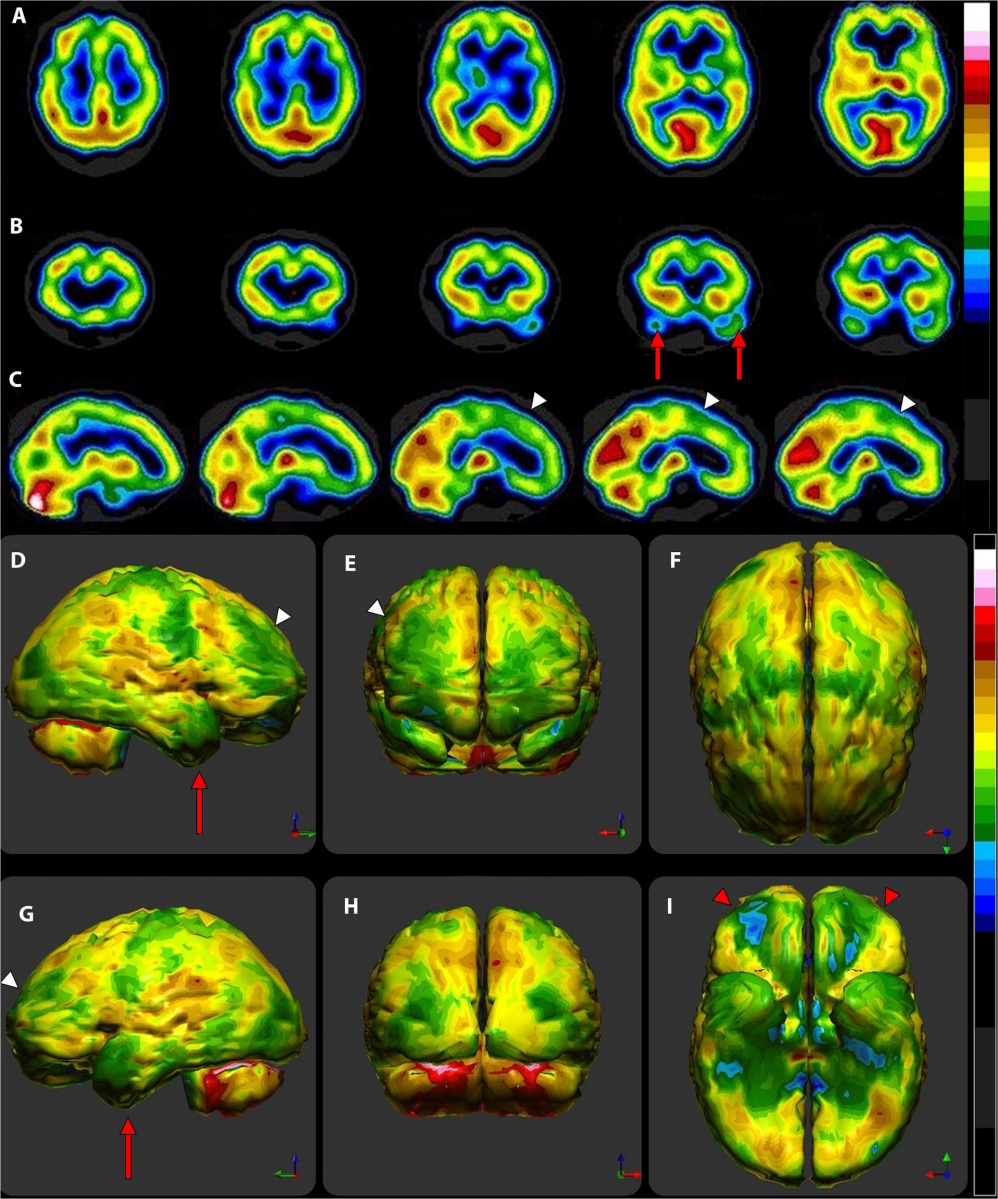
Tomograms and 3-D surface projections of perfusion SPECT scan showing mild TBI. **(A)** Selected transverse sections. **(B)** Selected coronal sections. Decreased perfusion in the temporal lobes is evident (red arrows) **(C)** Selected sagittal sections. Hypoperfusion is seen in the bilateral frontal cortex with greater involvement of the left side (white arrowheads). **(D–I)** 3-D surface projection views show the more extensive injury to the temporal (red arrows) and frontal cortices (white arrowheads) and scattered involvement of the bilateral parietal cortices. Marked hypoperfusion is seen in the orbitofrontal cortex (red arrowheads). **(D)** Left lateral view. **(E)** Anterior view. **(F)** Superior view. **(G)** Right lateral view. **(H)** Posterior view. **(I)** Inferior view with cerebellum removed. Hypoperfusion is evident in the orbitofrontal cortices bilaterally (red arrowheads). Color scale is the Ubiq40 color scale as described in Figure 5.

**Figure 10:**
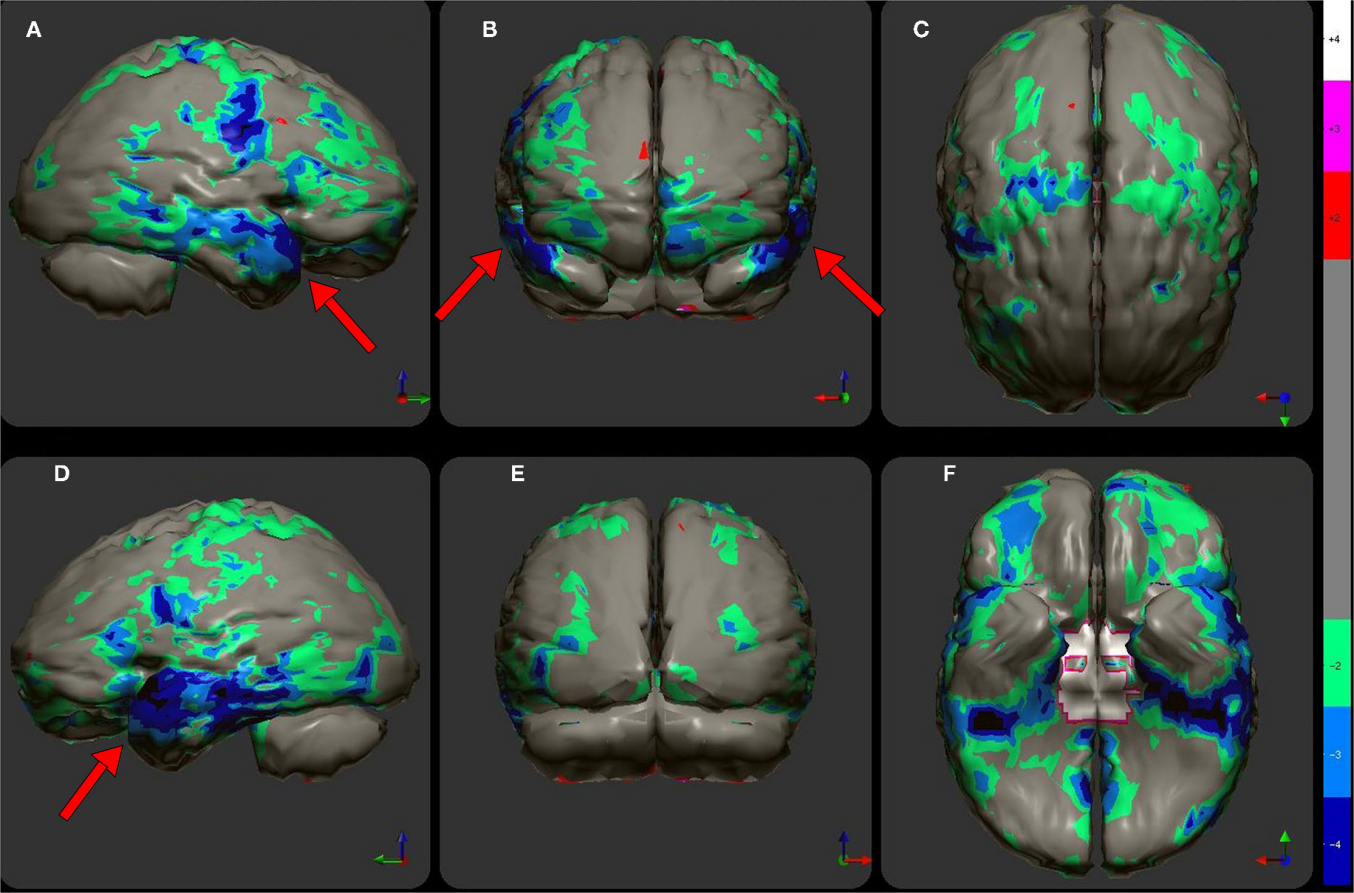
The patient's data is compared to a normative database (*N* = 68). A map of statistically significant differences can be generated using the Oasis software by Segami, Inc. Here, the color scale indicates gray for areas that do not differ significantly from the normative database. In contrast, areas of green, light blue, and dark blue represent areas of more than 2, 3, and 4 SD below the mean perfusion of the normative database, respectively. Statistically significant increases in perfusion are illustrated in the red color scale. Decreased perfusion in the bilateral temporal cortex and bilateral orbitofrontal cortex can be appreciated. Map is displayed in the following views: **(A)** right lateral, **(B)** frontal, **(C)** superior, **(D)** left lateral, **(E)** posterior, and **(F)** inferior with cerebellum removed.

The perfusion SPECT scan revealed hypoperfusion in the bilateral temporal lobes (left worse than right), bilateral frontal cortex (right worse than left), and scattered hypoperfusion in the bilateral parietal cortices (Figure 9). The scan is illustrated as both tomograms and a 3-dimensional map in Figure 9. Lastly, the scan was compared statistically to a normal database (Figure 10).

As discussed at length in Part I of this two-part series (2), perfusion SPECT is more sensitive than either CT or anatomical MRI for detecting TBI, including mild TBI as in the illustrated case. In longitudinal studies of TBI, SPECT identified lesions not found in CT or MRI in 100% of these studies (30, 125). Even in the acute setting, SPECT scans revealed lesions in 75% of patients who presented with amnesia symptoms, while CT scans were negative (126). Likewise, Stamatakis and colleagues (127) examined 62 patients with TBI using MRI and SPECT, which were performed within 2 weeks of injury. Using statistical parametric analysis, they found SPECT detected more lesions and more lesion volume than anatomical MRI. Ichise and colleagues (128) found SPECT scans more sensitive than MRI as well, with 79% of SPECT abnormalities lacking a concordant MRI lesion. Kinuya and colleagues (129) found SPECT detected hypoperfusion in 94% of cases wherein MRI scans were read as normal. These data call into question the position of several groups, the American College of Radiology, the American Psychiatric Association, and the American Academy of Neurology, which embrace the use of CT and MRI in the evaluation of concussive or traumatic injuries to the brain which constitute ***functional*** ***dyscracias***. A brain perfusion SPECT scan can not only help confirm or disprove TBI, but could give considerable aid in evaluating alternate or comorbid diagnoses, help evaluate the degree of injury, and aid the patients, themselves, understand the severity of their injury. The utility of SPECT perfusion neuroimaging in the evaluation of concussion and TBI is more extensively discussed in Part I of this two-part series (2).

### Situation 4

The fourth scenario involves a 23-year-old man with a prior history of social anxiety treated with sertraline, who suddenly developed an agitated state of motor restlessness and derealization. He described things seemed to be repeating themselves as though he was having recurrent déjà vu. The clocks seemed to be moving too slowly. He experienced difficulty understanding what others or television announcers were saying. His family described him as constantly moving—pacing and tapping on things. He repeatedly asked philosophical questions, such as “What is truth?,” What is good? What is evil?” He became increasingly confused—unsure if he was wearing a shirt or not—and paranoid. However, he was sleeping normally (8–9 h per night). He was seen in an emergency room. A neurological examination was normal. He had a negative CT, negative MRI, and negative electroencephalogram. Toxicology screen and laboratories were normal. He was eventually diagnosed with a manic episode and bipolar 1 disorder. He was prescribed olanzapine. While olanzapine improved the psychotic symptoms of derealization and paranoia, its efficacy did not answer the question of what caused the psychotic symptoms.

**Figure 11:**
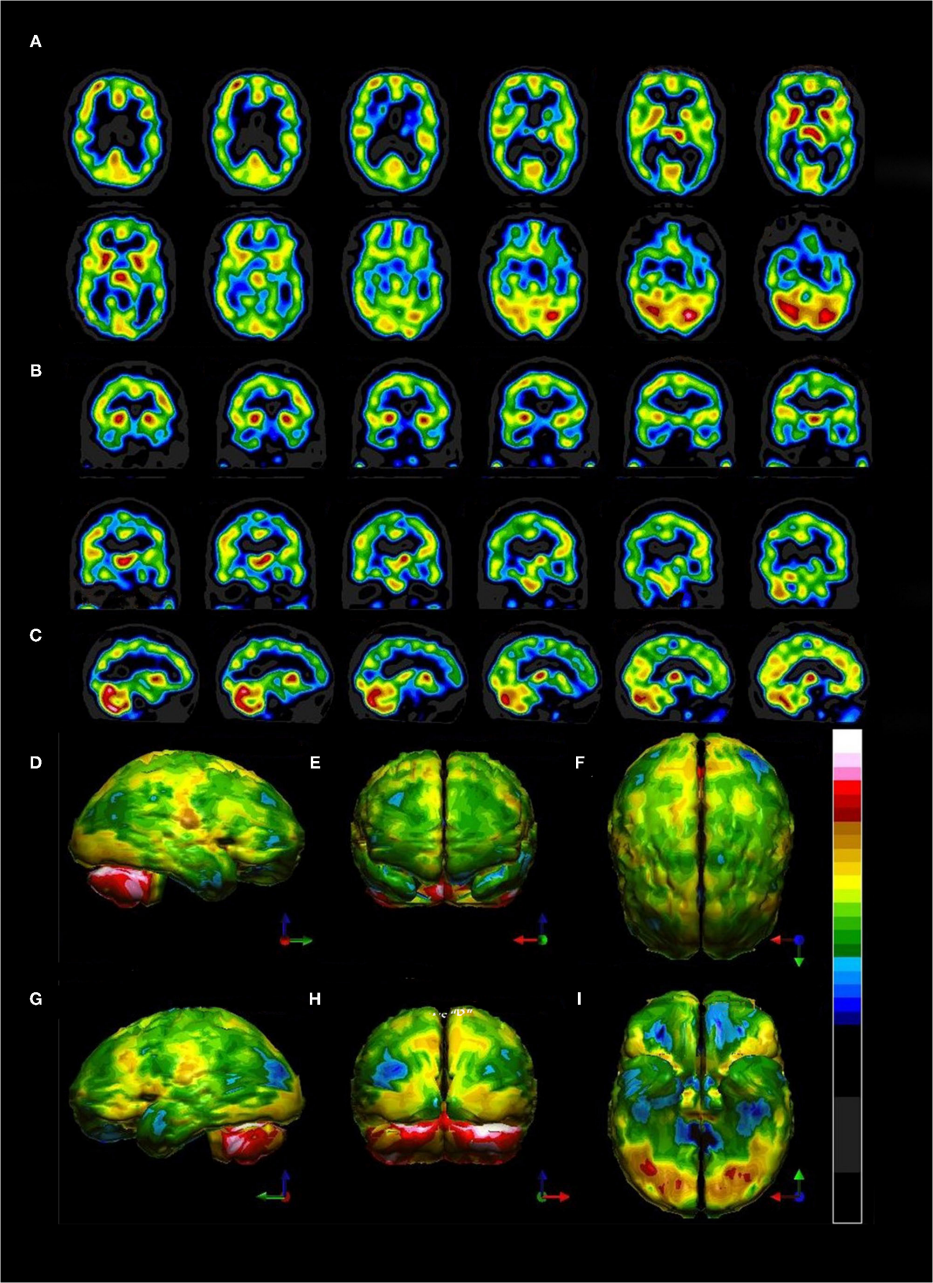
Tomograms and 3-D surface projections of perfusion SPECT scan showing diffuse hypoperfusion. **(A)** Selected transverse sections. Of note, the thalamus is neither hyper-perfused nor asymmetrical as is typically seen in bipolar disorder. **(B)** Selected coronal sections. The thalamus can be seen in a different orientation and is neither hyper-perfused nor asymmetrical. **(C)** Selected sagittal sections. **(D–I)** 3-D surface projection views show the highly difuse nature of the hypoperfusion involving frontal, temporal, parietal, and even occipital cortices. **(D)** Left lateral view. **(E)** Anterior view. **(F)** Superior view. **(G)** Right lateral view. **(H)** Posterior view. **(I)** Inferior view with cerebellum removed. Color scale is the Ubiq40 color scale as described in Figure 5.

**Figure 12:**
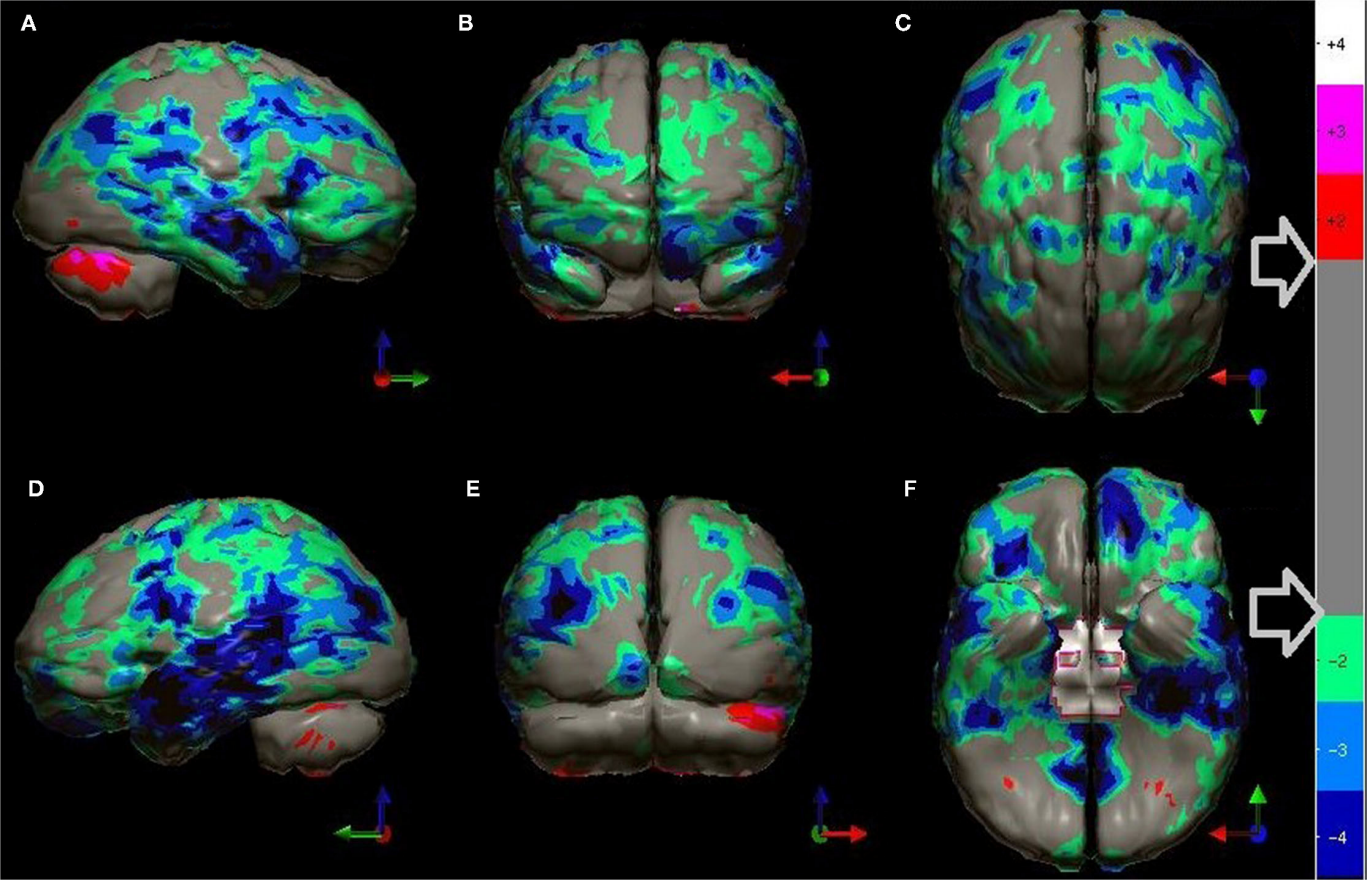
The patient's data is compared to a normative database (*N* = 68). A map of statistically significant differences can be generated using the Oasis software by Segami, Inc. The color scale is the same as in Figure 10. Diffuse decreased perfusion in all cortical areas can be appreciated. More extensive involvement of the bilateral temporal lobes is more clearly demonstrated with this map of statistical differences. Map is displayed in the following views: **(A)** right lateral, **(B)** frontal, **(C)** superior, **(D)** left lateral, **(E)** posterior, and **(F)** inferior with cerebellum removed.

A perfusion SPECT scan was performed. The typical findings of bipolar disorder were not evident. Specifically, the perfusion of the thalamus was not increased and was not asymmetrically perfused (30, 84, 129–131). Similarly, there was no evidence of increased cortical perfusion (30, 84, 130–132). Rather, the perfusion SPECT scan showed diffuse hypoperfusion (see Figures 11, 12). This finding is suggestive of a toxic brain injury or infection (2, 30, 82, 84). These perfusion SPECT findings prompted further questions (133).

Further history revealed no use of alcohol, marijuana, other substances of abuse, solvent sniffing, or other possible toxin exposures. The patient consumed city-provided water rather than well water, eliminating another possible source of toxins. History of travel revealed a trip to the Minnesota lakes region and multiple tick bites ~10 months prior to the index episode. The extensive involvement of the temporal lobes was consistent with findings in Lyme disease in the research literature (134, 135).

Blood tests were obtained from specialty labs. The patient had positive immunoglobulins for *Bartonella quintana* and for *Bartonella henselae*. Polymerase chain reaction (PCR) tests were also positive for these two infections. Northern blots were positive for both IgM and IgG for *Borrelia burgdorferi* based on Centers for Disease Control (CDC) research criteria (136) (IgM immunoblot positive for bands 23 and 41 with a negative Epstein-Barr test; IgG immunoblot positive for bands 28,30,45,58,66). Labs were negative for *Babesia microti*. Both *B. burdorferi* and *B. henselae* has been associated with psychiatric symptoms, including psychosis (137–142). Antibiotic therapy over the subsequent months brought resolution of symptoms and a repeat PCR was negative for both infectious agents. The patient was spared a lifetime diagnosis of bipolar disorder with the attendant psychopharmacological treatment and its potential adverse side effects.

## Discussion and Actionable Recommendations

Perfusion SPECT neuroimaging is a highly sensitive modality which has much higher contrast that CT or MRI, although it lacks the higher spatial resolution. This makes it ideal for looking at subtle (and not so subtle) changes in function over large areas using the one-off metric of local cerebral perfusion. SPECT scans now have much greater resolution, multiple normative databases, and sophisticated post-processing software. Moreover, the introduction of a new solid-state detector system utilizing CZT diodes will greatly increase the resolution of SPECT and reduce the radiation dose required. There are now numerous studies, often with quite large sample sizes, showing consistent and reproducible SPECT findings for a number of disorders. Given the major advances in our understanding of the SPECT functional neuroimaging findings for both neurological and psychiatric conditions as extensively reviewed in Part I of this two-part series, the position assumed in the wake of the TTASAAN report by both the fields of Neurology and Psychiatry is no longer tenable. As a result, the following recommendations are made for the revisions of the current policies and practices as they relate to perfusion SPECT functional neuroimaging:

1) Increase awareness of the actual current state of the art as iterated in Part I (2).
2) Replace assumptions about the inferior sensitivity and specificity of SPECT neuroimaging compared to PET, fMRI, diffusion tensor imaging, and MEG neuroimaging, particularly in the areas of dementia, TBI, seizure disorders, and neuropsychiatric indications with updated comparisons as elaborated in Part I (2) and as demonstrated in the clinical examples herein. This includes appreciating that SPECT neuroimaging does not need to be ***diagnostic*** as a freestanding technique. The clinical examples herein emphasized the role that SPECT neuroimaging plays in distinguishing among overlapping diagnoses, recognizing comorbidities, and prompting the “asking of better questions.”
3) Foster collaboration and communication between Nuclear Medicine physicians knowledgeable about perfusion SPECT neuroimaging and neurologists, psychiatrists, and other prescribers.
4) Enhance knowledge of the technical aspects of perfusion SPECT neuroimaging to improve understanding of the limitations and strengths of the procedure as elaborated herein.
5) Explore revision of Nuclear Medicine procedure guidelines to match the current state of the art as elaborated in Part I and Part II of this two-part series. This process has already begun with the publication of the new Canadian Association of Nuclear Medicine Guidelines for Brain Perfusion Single Photon Emission Computed Tomography (SPECT) (30).
6) Explore revision of Neurology practice guidelines to include the use of perfusion SPECT neuroimaging in the areas for which it has been shown to be most effective or on par with FDG-PET neuroimaging (e.g., seizure disorders, dementia, stroke). The case is made herein that SPECT is a potent tool in the evaluation of TBI and warrants inclusion in Neurology practice guidelines based on Level IIa evidence meeting the criteria for a Type A recommendation by the standards set forth in the TTASAAN report (1) and the moral impossibility of achieving Class I evidence.
  a. Additional guideline modifications to include the use of perfusion SPECT neuroimaging, when appropriate, in the differential diagnosis of dementia cases among amyloid-positive cases in the elderly (wherein false positives can occur) and in amyloid-negative cases of any age (as set forth in the clinical example herein).
  b. Additional guideline modifications to include the use of perfusion SPECT neuroimaging, when appropriate, in the differential diagnosis of Parkinsonian syndromes (as set forth in Table 1 and Figure 7 herein).
  c. Additional guideline modifications to include the use of perfusion SPECT neuroimaging, when appropriate, in the evaluation of toxic, infectious, or autoimmune encephalopathy. This may become particularly important in the growing population of patients with post-COVID fatigue or persistent encephalopathy (143).
7) Explore revision of Neurology and Psychiatry practice guidelines to recognize the advances in the state of the art of SPECT neuroimaging in the ***evaluation of*** ***neuropsychiatric indications***. As the Canadian Association of Nuclear Medicine Guidelines for Brain Perfusion Single Photon Emission Computed Tomography (SPECT) (30) describes, the diagnostic picture for all of neuroimaging is clouded due to the subjective and non-physiological bases of psychiatric diagnoses. This remains an unresolved issue.
  a. Include perfusion SPECT neuroimaging in the evaluation of encephalopathy, dementia, and toxic exposures (30).
  b. Recognize the value of SPECT in the monitoring or tracking of changes in brain function as a consequence of treatment (e.g., a biomarker) (130, 144, 145).
  c. Recognizing the value of SPECT in the uncovering and identification of co-morbidities.
8) Understand the basics of reading and interpreting a perfusion brain SPECT scan as elaborated herein and based on the findings summarized in Part I (2).

Perfusion SPECT scans require rigorous technique and correct adjustment of the equipment. An enormous variability in SPECT images results from technical inconsistency. One of the reasons why brain SPECT imaging is not prescribed more often is that prior encounters with sub-optimal images resulting from poor technique and lack of experience, have produced unhelpful findings. These negative experiences deter further use of SPECT scans and leads to fewer referrals. In part because of this dynamic and because of the parallel academic pressure to move to PET-based procedures, the attention, experience, and interest of nuclear medicine physicians has shifted away from SPECT imaging. In addition, the wholesale bias against perfusion SPECT scans discussed heretofore has stymied the clinical growth of this inexpensive, useful, and readily available procedure. The technical aspects of correctly performing and reading these scans has been provided to rectify this situation. The recent introduction of new camera technologies has made it possible to produce images that rival FDG-PET quality with lower radiation exposure and less cost.

## Conclusions

The path into the future will be paved with collaborations between neurologists, psychiatrists, and nuclear medicine physicians. Hopefully, enlightened clinicians in these fields will join together to deepen their clinical experience. Already progress has been made in identifying perfusion SPECT correlates in neurology and psychiatry as detailed in Part I (2). By applying the Food and Drug Administration's (FDA) Biomarkers, EndpointS and other Tools (BEST) glossary definition of a biomarker:

“a defined characteristic that is measured as an indicator of normal biological processes, pathogenic processes, or responses to an exposure or intervention, including therapeutic interventions.” (146).

we can see perfusion SPECT neuroimaging can serve as an objective biomarker of response to treatment (144, 145), comorbidity (30, 33, 82, 84, 133), inherently difficult symptoms to objectify, such as pain (147), and of diagnostic masqueraders as described herein and elsewhere (30, 33, 84, 133, 148).

## Author Contributions

DP and TH conceived the manuscript. DP and SD collaborated on initial drafts of the technical sections. TH, PC, and SD prepared the manuscript and the figures. All authors contributed to the article and approved the submitted version.

## Conflict of Interest

All authors are members of the International Society of Applied Neuroimaging (ISAN), a volunteer organization devoted to the understanding and appropriate clinical utilization of SPECT brain imaging. All authors volunteered their time in the research and writing of this manuscript. DP was Director of PathFinder Brain SPECT which is a clinical service corporation providing SPECT functional neuroimaging and had no research funding. He is deceased. TH is the president and principal owner of the Synaptic Space, a neuroimaging consulting firm. He is also CEO and Chairman of the Board of Neuro-Luminance Corporation, a medical service company. He is also president and principal owner of Dr. Theodore Henderson, Inc., a medical service company. He is also President of the Neuro-Laser Foundation, a non-profit organization. He is a member of and a former officer of the Brain Imaging Council Board of the Society of Nuclear Medicine and Molecular Imaging (SNMMI). Since 2017, he has served in the SNMMI Brain Imaging Outreach Working Group. Currently, he serves as president of the International Society of Applied Neuroimaging. TH has no ownership in, and receives no remuneration from, any neuroimaging company. No more than 5% of his income is derived from neuroimaging. SD is President of Good Lion Imaging LLC involved in the post-processing and display of functional brain scan data. PC has no commercial or financial relationships that could be construed as a potential conflict of interest.

## Publisher's Note

All claims expressed in this article are solely those of the authors and do not necessarily represent those of their affiliated organizations, or those of the publisher, the editors and the reviewers. Any product that may be evaluated in this article, or claim that may be made by its manufacturer, is not guaranteed or endorsed by the publisher.
